# In vitro analysis of the cytotoxic effect of two different sizes ITER-like tungsten nanoparticles on human dermal fibroblasts

**DOI:** 10.1016/j.heliyon.2023.e13849

**Published:** 2023-02-18

**Authors:** Lavinia Gabriela Carpen, Maria Adriana Acasandrei, Tomy Acsente, Elena Matei, Iulia Lungu, Gheorghe Dinescu

**Affiliations:** a National Institute for Lasers, Plasma and Radiation Physics, 409 Atomistilor Street, 077125, Magurele, Ilfov, Romania; b Faculty of Physics, University of Bucharest, 405 Atomistilor Street, 077125, Magurele, Ilfov, Romania; c Horia Hulubei National Institute for Physics and Nuclear Engineering, 30 Reactorului Street, 077125, Magurele, Ilfov, Romania; d National Institute of Materials Physics, 405A Atomistilor Street, 077125, Magurele, Ilfov, Romania

**Keywords:** Tungsten, Nanoparticles, Cytotoxicity, Fusion, Plasma

## Abstract

**Background:**

Based on the current configuration of the International Thermonuclear Experimental Reactor, tungsten (W) was chosen as the armour material. Nevertheless, during operation, the expected power and temperature of plasma can trigger the formation of W dust in the plasma chamber. According to the scenario for a Loss Of Vacuum Accident (LOVA), in the case of confinement failure dust is released, which can lead to occupational or accidental exposure.

**Methods:**

For a first evidence of potential risks, fusion devices relevant W dust has been produced on purpose, using a magnetron sputtering gas aggregation source. We aimed to assess the in vitro cytotoxicity of synthesized tungsten nanoparticles (W-NPs) with diameters of 30 and 100 nm, on human BJ fibroblasts. That was systematically investigated using different cytotoxic endpoints (metabolic activity, cellular ATP, AK release and caspase-3/7 activity) and by direct observation with optical and scanning electron microscopy.

**Results:**

Increasing concentrations of W-NPs of both sizes induced cell viability decrease, but the effect was significantly higher for large W-NPs, starting from 200 μg/mL. In direct correlation with the effect on the cell membrane integrity, high concentrations of large W-NPs appear to increase AK release in the first 24 h of treatment. On the other hand, activation of the cellular caspase 3/7 was found significantly increased after 16 h of treatment solely for low concentrations of small W-NPs. SEM images revealed an increased tendency of agglomeration of small W-NPs in liquid medium, but no major differences in cells development and morphology were observed after treatment. An apparent internalization of nanoparticles under the cell membrane was also identified.

**Conclusion:**

These results provide evidence for different toxicological outputs identified as mechanistic responses of BJ fibroblasts to different sizes of W-NPs, indicating also that small W-NPs (30 nm) display lower cytotoxicity compared to larger ones (100 nm).

## Graphical abstract

**Figure:**
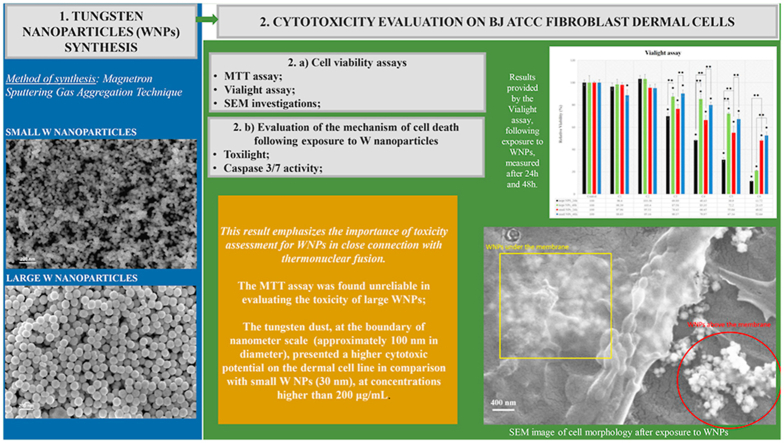

## 1 Introduction

The most exposed area of a tokamak, which must withstand long-term operation and demonstrate resistance to physical and chemical erosion, is the divertor. In this particular area, tungsten (W) has been chosen as armour material for its robustness, its elevated melting point and its low plasma sputtering yield [1]. The expected power and temperature of plasma, during operation, can trigger the formation of particles with variable sizes, ranging from tens of nanometers to hundreds of micrometers, that are called dust particles in the fusion community [2,3]. Apart from fusion plasma perturbation, this dust may pose security issues, depending on its chemical composition, the amount that is produced and the possibility that these particles to be released into the atmosphere [4,5]. The main concern is the possibility of generating an explosion, due to the violent oxidation of the dust after the contact with oxygen or water vapor, in case of accidental leakage of air or water [6,7]. Safety analysis of the ITER facility mainly aims to assess the risk of release of W dust in the environment, in case of accidental situations, such as Loss Of Vacuum Accident (LOVA) [8]. Even if High Efficiency Particulate Air (HEPA) filters are used to prevent the release of particles out of the vacuum chamber, these filters present minimum efficiency for particles between 50 and 300 nm [9]. So, in case of an uncontrolled release of these nanoparticles into the atmosphere, they may cause harmful consequences for ITER workers and for the environment safety. Therefore, the toxicity assessment of the dust resulting from a fusion reactor is of major importance [[9], [10], [11]].

Although research into the cytotoxic effect of W dust is already ongoing, the mechanisms and harmful effects are not yet fully and thoroughly known and these studies are in a limited number. W has been studied under different forms such as tungsten carbide (WC) alloys doped with cobalt (WC–Co) [[12], [13], [14], [15], [16], [17]], sodium tungstate (Na_2_WO_4_) [18,19], W oxide nanoparticles (WO_3_ NPs) [[20], [21], [22], [23], [24]] and metallic W [11].

In vitro exposure to metallic W microparticles has been shown to have no adverse effect at concentrations up to 100 μg/mL, but becomes significantly cytotoxic at higher concentrations [25]. Bolt et al. [26] have shown that W increases breast cancer metastasis when is used in medical devices. Studies of pure W induced toxicity are mostly focused on comparative studies between different materials, at macro- or micro-sizes, while the reports on this material toxicity at nanoscale size are limited [27].

In order to investigate the toxicity of W-based material, relevant dust particles were intentionally synthesized using different laboratory techniques, including milling, laser ablation and magnetron sputtering. All the produced particles show similarities with the samples collected in tokamak or observed in dusty plasmas setups, therefore could potentially be expected in ITER [10]. ITER-like milled W-NPs toxicity was assessed using the MucilAir® model [28], showing that these particles have a minor impact, in terms of toxic effects, cellular uptake and W transfer through the lung epithelium.

Also, in direct correlation with a potential case of LOVA, two types of ITER-like W-NPs (plasma sputtering or laser ablation), in their pristine, hydrogenated and tritiated forms were found to induce cytotoxic and epigenotoxic effects on BEAS-2B cells, irrespective of their synthesis method [11]. Increased oxidative stress could be a passway involved in W-NPs cytotoxicity and genotoxicity and the degrees of cytotoxicity is depending on the presence or absence of hydrogen on their surface. Unlike other studies [20], C. Uboldi et al. [11] found a much lower threshold concentration (1 μg/mL). Moreover, the W toxicity on normal human skin fibroblast cells was evaluated also in a book chapter [29]. Where a lower threshold concentration (up to 100 μg/mL) was found. At higher concentrations (up to 2 mg/mL), nanometric dust presents cytotoxic effects, as highlighted by scanning electron microscopy (SEM) [29].

The present study is intended to be supplementary to the characterization of the biological profile and the potential risk on human health of ITER-like W nanoparticles. By using the magnetron sputtering gas aggregation technique, W-NPs of two sizes (approximately 30 nm and 100 nm) were synthesized and analyzed to compare their toxicity on human skin fibroblasts cells. Herein, we investigated how the cytotoxic effects are related to the size of these nanoparticles. Cell-based assays are employed to address changes in cell metabolic activity, functional integrity and possible cell death mechanisms induced by W-NPs. For a first qualitative examination of W-NPs influence on cells, optical microscopy was used, and then we provide a thorough SEM investigation of cell morphology following W-NPs exposure.

## 2 Materials and methods

### 2.1 W nanoparticles synthesis

In this work, we used MSGA method for the synthesis of W nanoparticles, with dimensions of 30 nm and, respectively, 100 nm. A detailed description of the experimental setup can be found in Refs. [30,31]. Shortly, it consists of a cluster source based on MSGA, attached to a much larger particle deposition vacuum chamber (in which the particle collector is mounted). The cluster source consists of a cylindrical aggregation chamber with water-cooled stainless-steel walls, axially enclosing a magnetron sputtering gun, equipped with a 2” diameter W target (99.95% purity). Facing the target is mounted a conical shaped nozzle, ending in an exit aperture (diameters of 1.6 mm and 2.6 mm were used in this work), through which the communication with the vacuum deposition chamber is performed. The working gaseous atmosphere, entering the cluster source, is a mixture of Ar and H_2_ (∼5% partial pressure). Their mass flow rates are adjusted such as to have a pressure of ∼80 Pa in the cluster source for both diameters of the exit aperture. Due to the small diameters of the exit aperture, a pressure difference appears between the cluster source and the deposition chamber. Subsequently, the W-NPs obtained in the cluster source are transported by the gas flow, via the exit aperture, in the deposition chamber, where they are collected on substrates. During this work, the aggregation length (i.e. distance between the target and the exit aperture) was kept constant (90 mm). To support the discharge, a radiofrequency generator (13.56 MHz, applied power P_RF_ = 80 W) was used with an impedance matching box. In the following, the W-NPs are designated as small (30 nm) versus large (100 nm) on the basis of the average particle diameters determined from SEM images.

### 2.2 Nanoparticles characterization after synthesis

After synthesis, observation of W-NPs morphology and measurement of particles’ size were obtained using SEM with a Gemini 500 equipment from Zeiss (Oberkochen, Germany). Size distribution of the nanoparticles and the average size were processed using ImageJ software [32].

The physical and chemical stability of the W-NPs in the biological medium were assessed by the Dynamic Light Scattering (DLS) technique using the Horiba Scientific SZ-100 equipment, at 25 °C. The samples were dispersed and diluted in the same manner as for cytotoxicity studies.

### 2.3 Preparation of W-NPs dispersions

W-NPs were first collected by scratching from the glass substrate, weighed and added in phosphate - buffered saline (PBS). To ensure particles dispersion, stock W-NPs suspensions (10 mg/mL) were sonicated using a probe sonicator (Ultrasonic Homogenizer, model 300VIT). Subsequently, the stock solutions were sterilized by autoclavation for 15 min, at 136 °C. Stock solutions were diluted to different concentrations (1–1000 μg/mL) in complete cell culture medium, which were used further for toxicological experiments. This range of concentrations (Table 1) was selected based on our previous study [29].

**Table 1:** Dilutions based on stock concentration.

Sample	The concentration of the suspensions (μg/mL)
C (Control sample)	–
C1	1
C2	10
C3	100
C4	200
C5	400
C6	1000

### 2.4 Cell culture and exposure to W-NPs

For biological testing, we used BJ human skin fibroblasts cultures (ATCC™ CRL-2522) which were derived from an original ATCC batch. Fibroblasts were cultured in Eagle's Minimum Essential Medium (EMEM) supplemented with 10% Fetal Bovine Serum (FBS), 100 U/ml penicillin and 100 μg/mL streptomycin. Cells were incubated at 37 °C in a humidified atmosphere of 5% CO_2_.

For toxicity assays, BJ cells were inoculated at 5 × 10^4^ cells/mL in 96-well plates and incubated for 24 h. Then, the medium of each well was removed and 100 μL of the nanoparticle suspensions in different concentrations in the cell culture medium was added to wells. In untreated cells, only a complete medium was added. The cells were incubated for different exposure times.

### 2.5 Cytotoxicity assays

After treatment, W-NPs suspensions were removed and the cytotoxic effects were tested by methods based on different viability parameters: metabolic activity of viable cells by a colorimetric assay (MTT, Serva), adenosine triphosphate (ATP) content of the cells (ViaLight Plus BioAssay, Lonza) and cellular necrosis indicated by adenylate kinase (AK) release (Toxilight™ assay, Lonza). Moreover, as an index of apoptotic entry, increases of caspase 3/7 activity in BJ cells were measured (Apo-ONE Homogeneous Caspase-3/7 Assay, Promega). The measurements are performed according to the working protocols offered by the manufacturing company. The data were taken with the help of Mithras microplate reader spectrophotometer, Berthold Technologies.

**MTT.** This method is based on the reduction of a tetrazolium salt (3-[4,5-dimethylthiazole-2-yl]-2,5-diphenyltetrazolium bromide - MTT compound) by the mitochondrial oxidoreductase enzymes, which are found in viable cells. The MTT formed formazane in living cells has a maximum absorbance in the range of (550–600) nm, which is proportionally related to cells viability. Cells were cultivated and treated as described in Section 2.4*.* After the treatment, the medium from the wells was thrown, the MTT mixture was added and the cells were kept 3 h at 37 °C in a humidified and 5% CO_2_ atmosphere. At the end of this incubation, the supernatants were discarded and the dark blue formazan crystals were dissolved using 100 μL DMSO added in each well. The absorbance was measured at 590 nm with a reference wavelength of 690 nm, by a plate reader. The absorbance at the reference wavelength (690 nm) was automatically subtracted from the absorbance at the test wavelength. (590 nm). Samples without cells, but with the medium in which W-NPs are dispersed, were used as blank control. After blank subtraction, results were expressed as mean percentage decrease relative to unexposed control. Control values were fixed at 100% viability.

**ViaLight assessment** was performed in order to determine the intracellular level of ATP, which is a measure of the functional integrity of living cells and it reflects their metabolic function. Basically, it detects, as a measure of cell viability, the bioluminescence of luciferin arising from its interaction with cellular ATP (reaction catalyzed by the luciferase enzyme). In order to achieve these results, the ViaLight Plus BioAssay was used according to the manufacturer's instructions (Lonza). Briefly, cells were plated in 96-well plates and treated with the various W-NPs concentrations, as described under MTT assay. After W-NPs exposure, 50 μl of cell lysis reagent was added to each well and left for 10 min at room temperature to extract ATP from cells. After ATP extraction, 100 μl of ATP monitoring reagent plus was added to each well and left for 2 min to equilibrate. Luminescence was checked on a microplate reader at 1s integration. The cell viability was determined in comparison to similarly processed untreated control cells (100% cell viability).

**ToxiLight.** To analyze whether exposure to W nanoparticles could result in cell death caused by deterioration of cell membrane integrity (cytolysis), the bioluminescent ToxiLight™ Non-Destructive Cytotoxicity BioAssay Kit (Lonza Rockland, Inc., Rockland, ME, USA) was used, according to the manufacturer's instructions.

This test measures the release of AK into the extracellular environment, from damaged cells. AK is a protein that is present in all eukaryotic cells and is rapidly released into the culture medium following damage to the cell membrane. The intensity of the emitted light is linearly dependent on the AK concentration. After the incubation period, to achieve a total amount of AK control (positive control) a Toxilight™ 100% lysis reagent was added in untreated controls. The use of the lysis reagent will intentionally damage the cell membrane in the positive control samples and this is proportionally related to the total number of cells. Equal volumes of the tris acetate buffer were added to the sample wells. After 10 min incubation, to ensure complete lysis in the control, 20 μl of cell supernatant were transferred to a white 96-well microtiter plate. Then, 100 μl of the detection reagent was added to each well. After 5 min, the luciferase signal was detected by use of the microplate reader. The cytotoxic response was presented in percentage reported to the total number of cells in each sample.

**Apo-ONE® Homogeneous Caspase-3/7 Assay**. The assay provides the necessary reagents for measuring the activities of caspase-3 and -7, two effector caspases involved in a late stage of apoptosis. After treatment, Apo-ONE® Homogeneous Caspase-3/7 reagent is prepared according to the manufacturer's protocol. Briefly, cells were incubated with W-NPs at the studied concentrations for 16 h. After this time, 100 μL of reaction mixture (profluorescent substrate in lysis buffer) was added in each well, the contents of wells were gently mixed and incubated at room temperature for 2 h. The fluorescence was then measured using 499 (excitation) and 521 nm (emission) with the microplate reader and adjusted in relation to the cell densities (total number of cells per well). The total number of cells in each well is correlated with the cell's nuclear material (DNA). Thus, the loss of nuclear material was performed by labeling with Hoechst 33342 fluorophore (Invitrogen). The values measured by labeling the nuclear DNA with Hoechst (5 μg/mL, 355 nm excitation and 460 nm emission), which correlates with the total number of cells in each well, were further used in normalizing the values of caspase-3/7 activity [33,34]. The results were presented in percentage comparatively to untreated samples.

### 2.6 Cell morphology

To determine by qualitative means the influence of W-NPs on morphological changes in BJ dermal fibroblasts, images of the cells exposed to different concentrations of W-NPs were obtained using an inverted optical microscope (CKX31SF, Olympus, Japan).

SEM was used to obtain complementary information both on cellular morphology changes induced after cells exposure to W-NPs and on prospective internalization of these nanoparticles under the BJ cell membrane surface. Briefly, the BJ cells were seeded onto coverslips and incubated for 24 h to adhere, followed by exposure to W-NPs for another 24 h. Then, the samples were fixed in 2.5% glutaraldehyde in PBS, followed by dehydration with ascending series of water-ethanol solutions (10, 30, 50, 70, 90 and 100% for 10 min each) and HMDS drying (100% for 10 min). After that, HMDS was decanted, and the samples were left under a hood to air-dry at room temperature. Afterward, the samples were coated with a thin layer of 5 nm of gold.

The SEM investigations on cellular morphology were performed using a Zeiss EVO 50 XVP equipment with LaB6 electron gun, in the secondary electron mode. The cell morphology after WNPs exposure was also investigated without the thin layer of gold, in order not to alter the morphological properties. These images are added as Supplementary Material.

### 2.7 Statistical analysis

The cytotoxicity experiments were performed in three independent tests. The units of absorbance/fluorescence/luminescence obtained in the cytotoxicity assays were converted to percent of controls and are presented as mean ± SD. All these results were statistically analyzed using one-way ANOVA (due to the fact that we had normally distributed data - evaluated using Anderson-Darling). Significant differences between means were determined using the Tukey test, based on a confidence level at P ≤ 0.05. All these data interpretations were performed using OriginLab.

## 3 Results and discussion

### 3.1 ITER-like W-NPs physico-chemical characterization

**Fig. 1:**
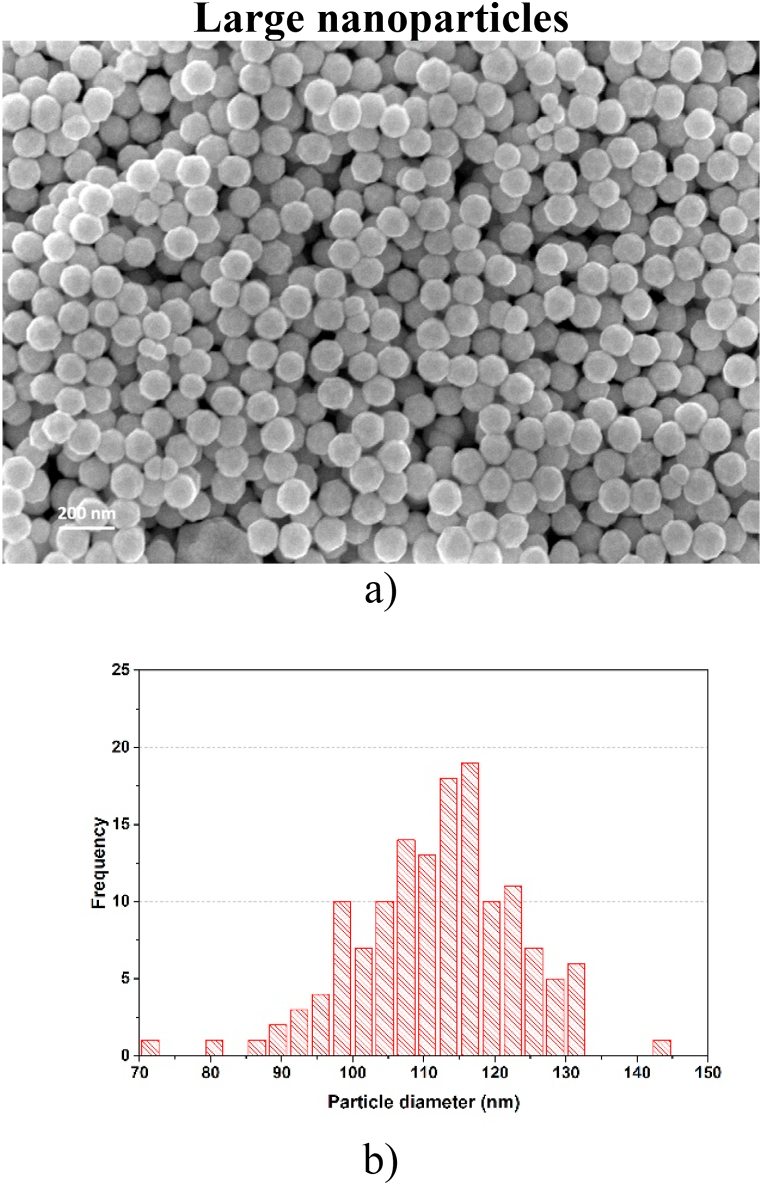
a) SEM image of deposited W-NPs using a 1.6 mm nozzle (Large nanoparticles) [29]; b) Size distribution histogram for large W-NPs population.

**Large nanoparticles.** These W-NPs were synthesized by the MSGA technique using a 1.6 mm nozzle and the sample consists of nanoparticles with a diameter size around 100 nm. From SEM images, shown in Fig. 1(a), the morphology of the large synthesized particles was observed to be spherical. The corresponding size distribution histogram was calculated from data obtained using ImageJ software [Fig. 1(b)]. It shows that this sample contains large nanoparticles with diameters from 72 nm to 147 nm, the diameter with the highest frequency was obtained for 110–120 nm.

**Fig. 2:**
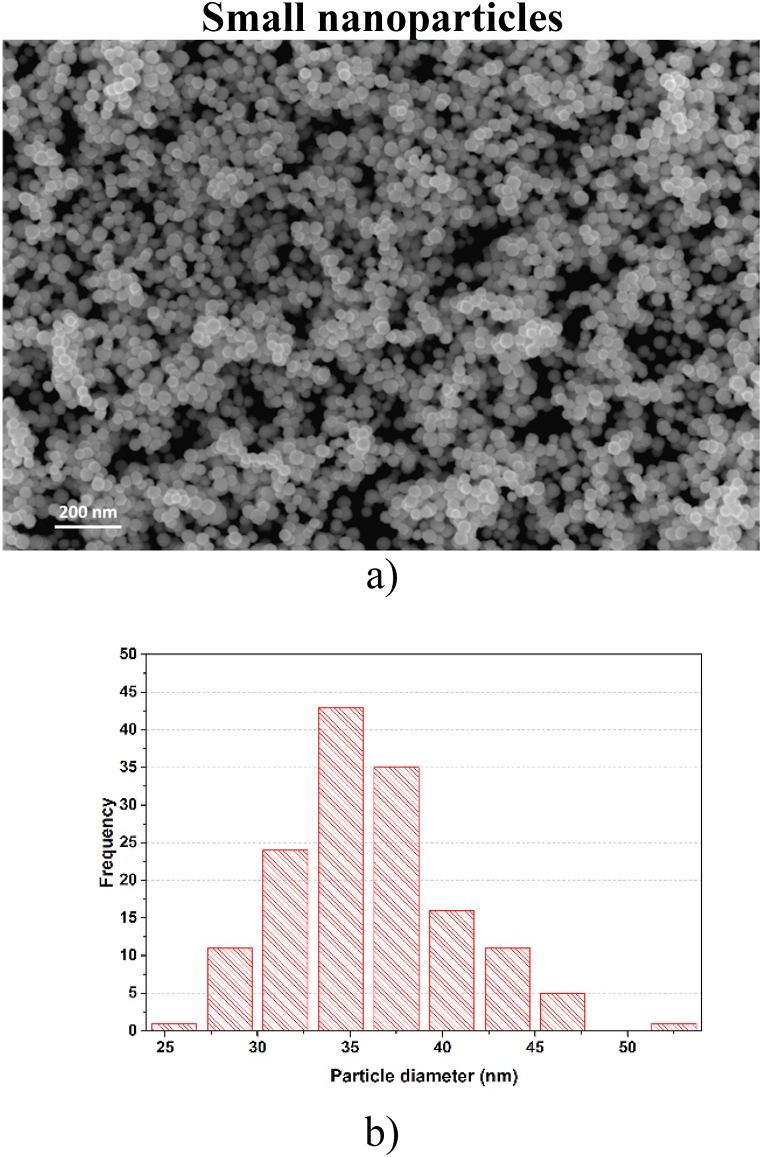
a) SEM image of deposited W-NPs using a 2.6 mm nozzle (Small nanoparticles); b) Size distribution histogram for small W-NPs population.

**Small nanoparticles.** When argon and hydrogen were introduced into the discharge (MSGA method) and by using the 2.6 mm nozzle, there were obtained W-NPs with a diameter size around 30 nm. The morphology and size of the nanoparticles were analyzed from the SEM images shown in Fig. 2(a). The synthesized particles have a spherical morphology (Fig. 2(a)), with a distribution of diameters in the range of 25–52 nm (Fig. 2(b)). The diameter with the maximum frequency was obtained for small nanoparticles within the range of 34–36 nm.

**Fig. 3:**
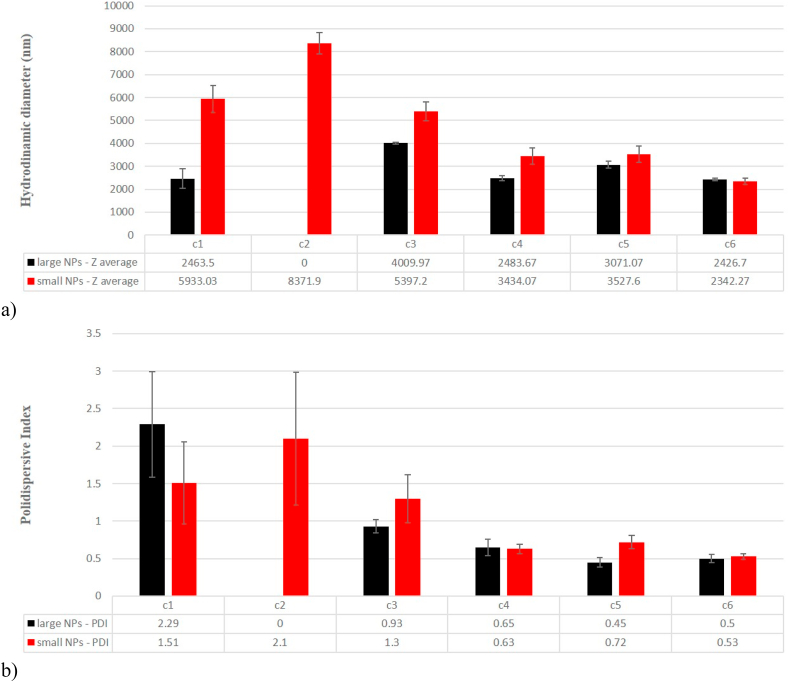
Evaluation of the hydrodynamic diameter (a) and the PDI polydispersity index (b) for the large and small nanoparticle samples, according to their suspensions concentration that are used in this study.

**Aggregation of W-NPs in culture media.** DLS was used to determine the average hydrodynamic diameter and polydispersity index (PDI) of each batch of nanoparticles, when dispersed in liquid media. We observed that both small and large synthesized W-NPs have a high tendency to agglomerate in the liquid medium, being more accentuated in the case of small nanoparticles, where a maximum hydrodynamic diameter (9 μm) is observed (Fig. 3(a)). At low W-NPs concentrations (≤100 μg/mL) the hydrodynamic diameter exceeds 5 μm for the samples containing small NPs and, respectively, 2 μm for the large ones. Regarding the average hydrodynamic diameter and polydispersity index (PDI) for c2 concentration, in the case of large nanoparticles, due the limitations of the equipment and based on the tendency of this material to agglomerate in liquid medium, this information was not detected. At higher concentrations (200, 400 and 1000 μg/mL) the hydrodynamic diameter decreases to a minimum of 2.5 μm, for both types of samples. In Fig. 3(b) PDI variation is presented, highlighting that W-NPs in suspension form agglomerates of different sizes. Up to 100 μg/mL, PDI values were bigger than 0.7, which indicates that both small and large W-NPs samples have a very broad particle size distribution and also that the samples are not easily analyzed by DLS. At higher concentrations (200, 400 and 1000 μg/mL) PDI decreases to about 0.5.

### 3.2 Evaluation of the cytotoxic effects

#### 3.2.1 MTT assay

**Fig. 4:**
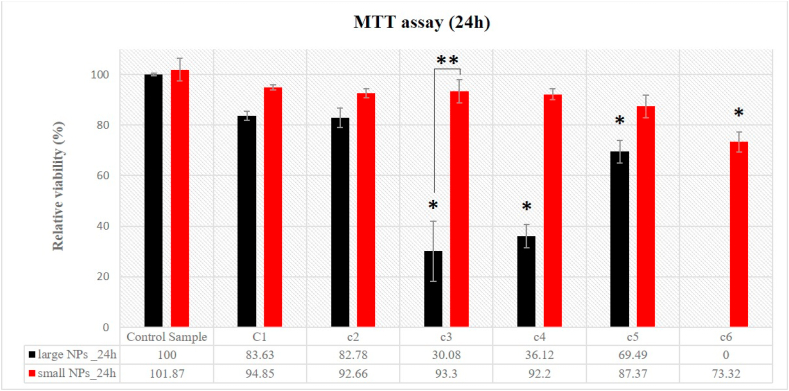
Cytotoxicity assessed by MTT assay. It highlights the metabolic activity of BJ cells following 24 h exposure to small or large W-NPs, at different concentrations. The means are statistically different starting with c3 concentration, for large NPs. Regarding small nanoparticles, just for c6 concentration, the means are statistically different, in relation with the control sample; (*) Denotes statistically significant difference, in relation to the control sample values; (**) Denotes statistically significant difference between small and large W-NPs (P-values⩽0.05).

The effects of the two types of W-NPs, differing in size, on BJ cells viability were examined after 24 h exposure, by using the MTT assay (Fig. 4). Consistent with our previous findings [29], MTT results obtained for large nanoparticles could be physically relevant only at W-NPs concentrations lower than 100 μg/mL, where no significant viability decrease was seen at 1 and 10 μg/mL concentrations. Starting with c3 concentration (100 μg/mL), the reduction of MTT compound by large W-NPs, in a concentration-dependent manner, was observed in the cell-free wells (blanks). This result indicates an interference of large W-NPs with MTT assay, which strongly overestimates the cytotoxic effect of the large W-NPs. The MTT results could be erroneously interpreted as a rapid decrease in cell viability with increasing concentrations of large W-NPs, which looked apparently in contrast with the qualitative observation of microscopic images of the cells. Therefore, under our study conditions, MTT assay proved to be unreliable for testing cytotoxic effects induced by large W-NPs.

**Fig. 5:**
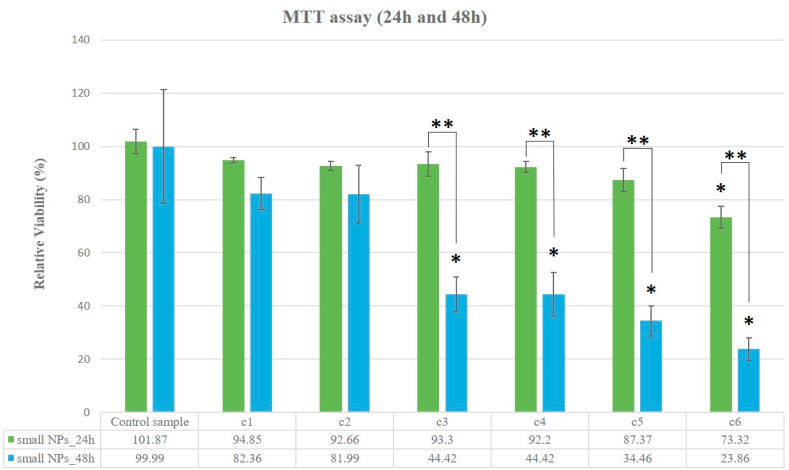
Cytotoxicity assessed by MTT assay. Comparison of the metabolic activity of BJ cells following 24 h and 48 h exposure to small W-NPs, at different concentrations. The means are statistically different starting with c6 concentration, after 24 h exposure, and c3 concentration, after 48 h exposure, respectively; (*) Denotes statistically significant difference, in relation to the control sample values; (**) Denotes statistically significant difference between 24 h and 48 h exposures (P-values⩽0.05).

Lack of an apparent interference of small W-NPs with MTT compounds stimulated us to further examines by MTT assay the time-dependent cytotoxicity induced by small W-NPs, looking at the metabolically active BJ fibroblasts after 48 h of treatment. In the case of small nanoparticles (30 nm), MTT assay demonstrated that the cytotoxic effect was clearly concentration- and time-dependent for BJ cells (Fig. 5). According to MTT data after 24 h exposure, there is no significative decay in cell viability with increasing NPs concentrations, up to c6 (1000 μg/mL). However, we would point out that the maximum exposure concentration (c6) of small W-NPs significantly decreased the cell viability to 73.32%, after 24 h. But, after 48 h exposure, small W-NPs significantly decreased the viability of BJ cells starting with c3 concentration (100 μg/mL). Therefore, we can emphasize that there is a significant decrease in cell viability, for small NPs, after 48 h of exposure, at a concentration starting with 100 μg/mL, in contrast to 24 h exposure.

#### 3.2.2 Vialight assay

**Fig. 6:**
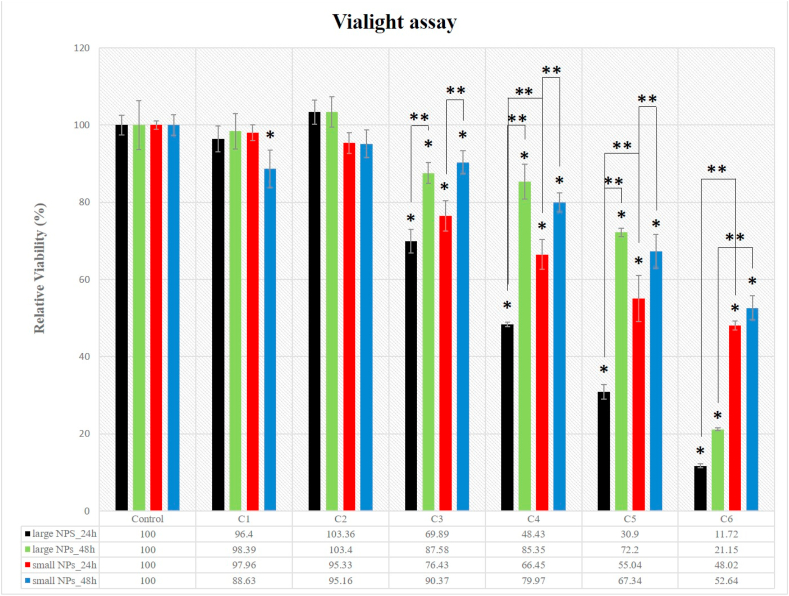
Cell viability measured by Vialight assay. It highlights BJ cells viability in terms of cell-ATP level, following exposure to W-NPs, measured after 24 h and 48 h. Regarding both 24 h and 48 h exposure, the means are statistically different starting with c3 concentration for small NPs and large NPs, in relation with the control sample; (*) Denotes statistically significant difference, in relation to the control sample values; (**) Denotes statistically significant difference between small and large W-NPs and between 24 h and 48 h exposure (P-values⩽0.05).

The Vialight assay, known to be much more sensitive than MTT assay, offers a testing method for cell viability by determining the intracellular ATP content of the cells. As observed in Fig. 6, increasing concentrations of W-NPs of both sizes induced a decrease in cell viability. The 24 h treatment with small nanoparticles induced a decrease of cell viability from 97.9% at 1 μg/mL to 48.02% at 1 mg/mL, with a significant decrease of the viability (76.43%) at 100 μg/mL. In the case of large W-NPs, viabilities decrease from 96.4% at 1 μg/mL to 11.72% at 1 mg/mL, with a significant effect starting also from 100 μg/mL (viability ∼ 69%). Therefore, 24 h exposure to increasing concentration of W-NPs of both sizes does not appear to have cytotoxic potential up to a concentration of 100 μg/mL. As well important is the finding that from 200 μg/mL (c4) onward, Vialight assay indicates significantly higher cytotoxic effect of larger W-NPs as compared with the small ones. By doubling the treatment time (48 h) and comparing with the results from 24 h, a significantly increase in cells viabilities is observed for 100 μg/mL (c3), 200 μg/mL (c4) and 400 μg/mL (c5) concentrations, both for small and large W-NPs. The results of such comparison must be further interpreted with caution as ATP levels do not always correlate with the number of viable cells, as will be referred to below, in the Discussions section.

### 3.3 The evaluation of the cell death mechanism

#### 3.3.1 Cell membrane integrity

**Fig. 7:**
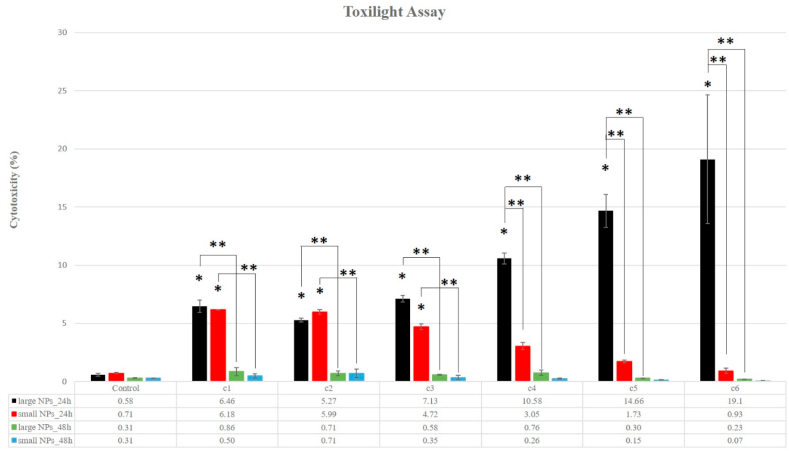
Cytotoxic effects of W-NPs on BJ cells measured by AK release via Toxilight assay. Results were obtained following 24 h and 48 h of exposure to small and large WNPs. After 24 h, the means are statistically different starting with c1 concentration, for both small NPs and large NPs, in relation with the control sample; (*) Denotes statistically significant difference, in relation to the control sample values; (**) Denotes statistically significant difference between small and large W-NPs and between 24 h and 48 h exposure (P-values⩽0.05).

The cytotoxicity, in direct correlation with the cytolysis process (the integrity of the cell membrane), was measured by the ToxiLight test and it is presented in Fig. 7. We found that the cytotoxicity after 24 h of treatment was low for all samples of cells (<10%) when treated with concentrations of W-NPs (both small and large) lower than 100 μg/mL, with no significant difference between the two sizes. Only higher concentrations (>100 μg/mL) of large W-NPs appear to have further increased the cytotoxic effect. After 48 h of treatment, AK release decreased drastically at all concentrations used, for both small and large W-NPs. Thus it seems that cell membrane damage could be the main mechanism by which cell death occurs, at least after large W-NPs exposure.

#### 3.3.2 Apoptosis cell death mechanism

**Fig. 8:**
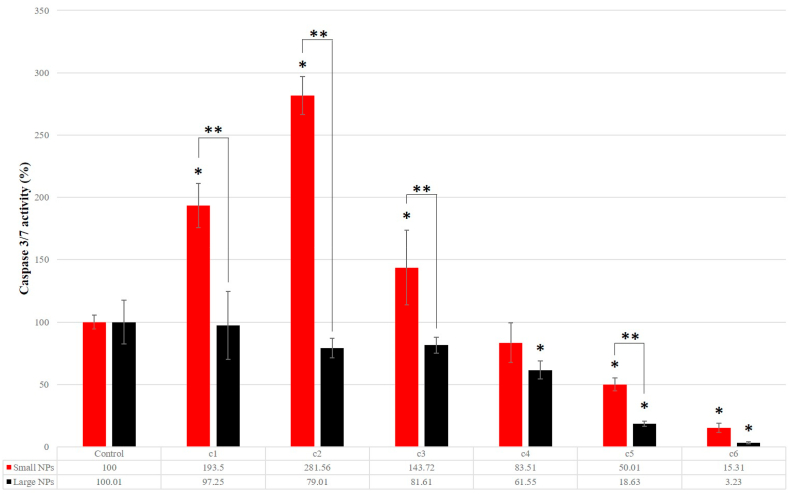
Caspase 3/7 activity in response to 16 h of W-NPs exposure to BJ cells. Results are presented in percentage comparatively to untreated samples and were calculated based on triplicated values. For small W-NPs, the means are statistically different for all investigated concentrations, except c4; for large NPs, the means are statistically different starting with c4. (*) Denotes statistically significant difference, in relation to the control sample values; (**) Denotes statistically significant difference between small and large W-NPs (P-values⩽0.05).

As an indicator of the apoptosis-related process, the activation of the cellular caspase 3/7 was measured after 16 h of treatment with W-NPs. Overall, small W-NPs induced significantly higher levels of caspases-3/7, as compared with those induced by large W-NPs (Fig. 8) and this increase is observed when cells are treated with concentrations lower or equal than 100 μg/ml. At higher concentrations, for both investigated samples, almost complete inhibition of caspase activity is observed.

### 3.4 Microscopy investigations

#### 3.4.1 Optical microscopy

**Fig. 9:**
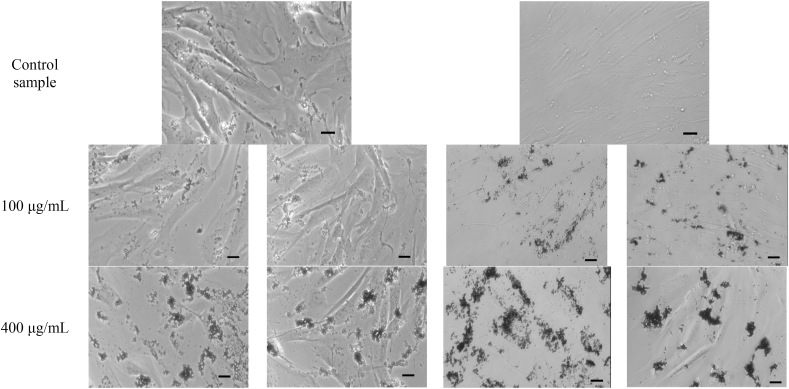
Optical microscopy investigations of BJ cells at 20× magnification. The scale bar is 30 μm.

Visual inspection of the BJ fibroblast cells (Fig. 9) apparently indicates a less confluent layer of cells when treated with large nanoparticles, than with small nanoparticles. This might signify a slightly higher cytotoxic effect induced by large nanoparticles, which is in agreement with the viability results (Vialight assay). In addition, one can see that after particle treatment for 24 h, agglomerated W-NPs are present on the entire well surface, consistent with their relative large sizes. After another 24 h, these agglomerates become un-evenly distributed across the surface of the well, suggesting most likely a factor related to cells movement.

#### 3.4.2 SEM investigations

**Fig. 10:**
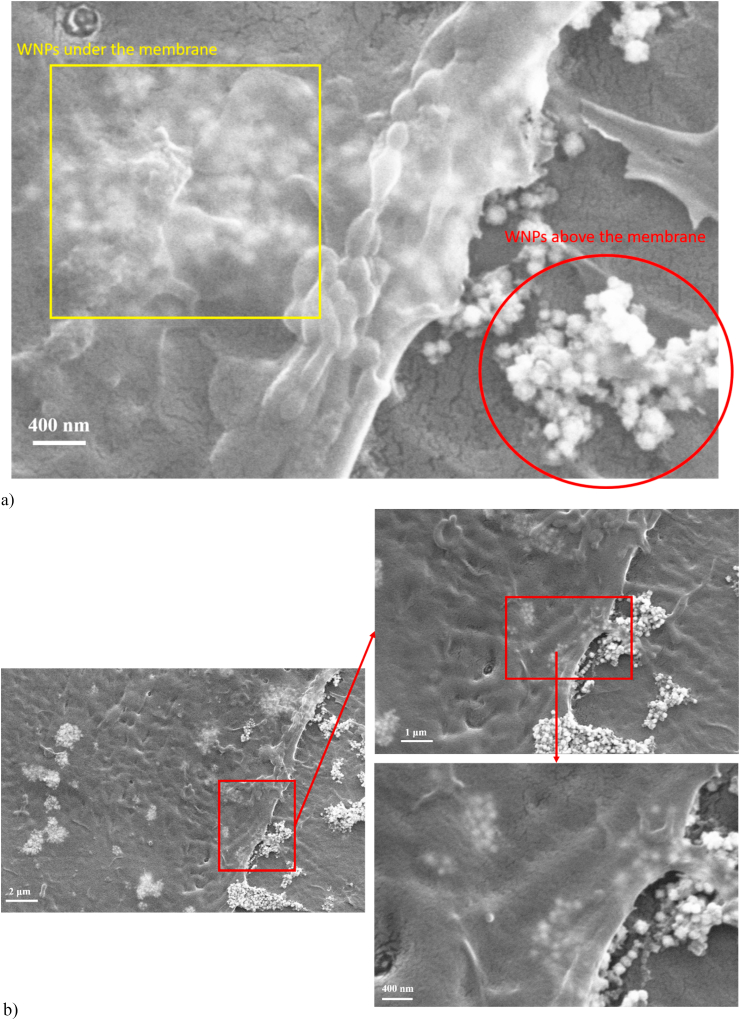
SEM images, at various magnifications, that capture the effect of large W-NPs (100 μg/mL) on BJ fibroblasts morphology. a) A more detailed representation where the possible internalization is highlighted (yellow frame) and also the agglomerates of NPs deposited on the surface of the cell body (red circle); b) SEM images at different magnifications, with focus on cell-body edge, illustrating cell-particle interaction.

**Fig. 11:**
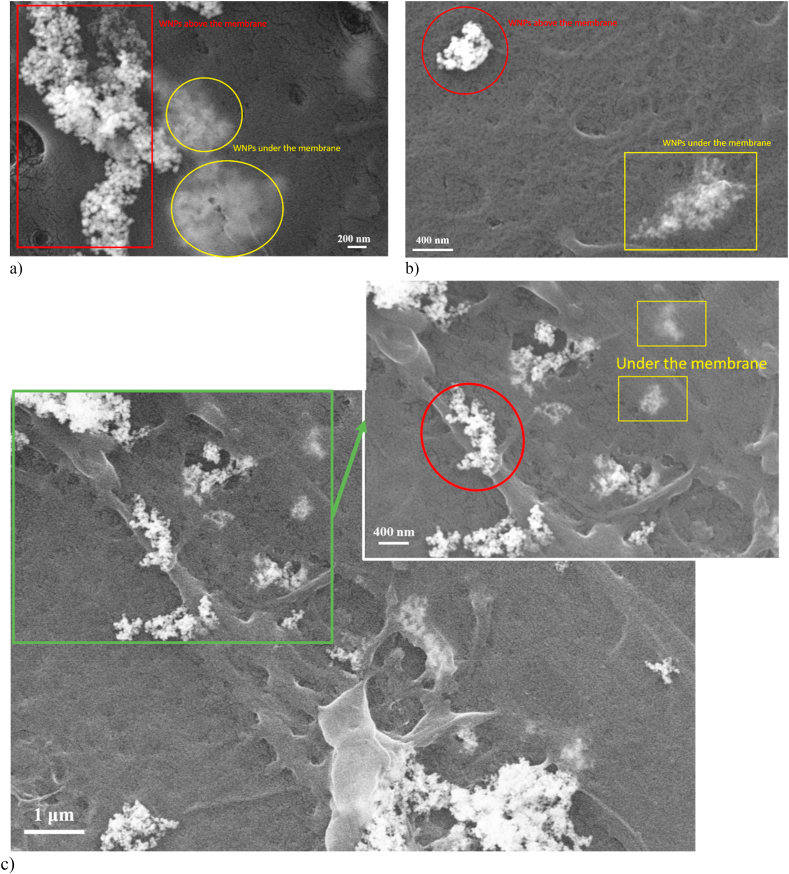
SEM images that reveal small W-NPs interaction with BJ cells. a) Aspects of cell morphology; b) A possible subsequent uptake into the cell body (outline with yellow frames), and the NPs agglomerates deposited on the surface of the cell body (red frames); c) Distribution of small W-NPs agglomerates and cells behavior after exposure.

In order to determine if there are effects on the morphology of BJ cells, SEM images were obtained after 24 h treatment with different concentrations of large and small W-NPs (Fig. 10, Fig. 11, respectively). Firstly, SEM images revealed an increased tendency of agglomeration for both small and large nanoparticles, these agglomerates being distributed on the entire cell surface. In particular, at the highest W-NPs concentration (1000 μg/mL), the agglomerates were observed to settle on the cells surface, more noticeable when treated with small nanoparticles (see Supplementary Material).

Overall, there are no major differences in cell development and, implicitly, in cell morphology compared to the control sample (Supplementary Material). The cells retained their shapes after 24 h treatment, which indicates that W-NPs did not severely affect cells integrity. However, a comparison of the confluency reached by cells following treatment, indicates that small W-NPs are less cytotoxic on BJ fibroblasts than large W-NPs, likely due to their higher tendency to agglomerate in the liquid medium.

Similar to our prior study [29], we identified an apparent internalization of nanoparticles under the cell membrane. Fig. 10, Fig. 11 and 11(b) and 11(c) highlight aspects of the way in which the nanoparticles could be internalized by cells (yellow frames) and also the agglomerates deposited on the surface of the cell body (red frames), for both small and large NPs. Besides, Fig. 10, Fig. 11, at various magnifications, outline how cell morphology is affected after 24 h exposure to 100 μg/mL of large or small W-NPs, respectively.

## 4 Discussion

The aim of our study was to provide an evaluation of cytotoxic effects that ITER-like nanoparticles, at different dimensions, may induce on a human skin fibroblast model and to explore possible cell death mechanism pathways, in direct relation with the behavior of these NPs in biological medium. We focused our attention on the BJ human skin fibroblast cell line because skin interaction is one of the principal routes of ITER workers' exposure to W dust. Until the ITER reactor becomes operating to have ITER-derived WNPs, ITER-like nanoparticles were synthesized using a cluster source based on the MSGA method. This method has the advantage of being similar with the particle production in fusion reactors and also it assures a high chemical purity of the obtained nanoparticles using a small number of precursors [30,[35], [36], [37]].

Using different experimental synthesis conditions, two sizes of spherical nanoparticles were obtained with average diameters of ca. 30 nm (small W-NPs) and 100 nm (large W-NPs). After dispersing the W-NPs in culture media, at the concentrations that were investigated, the DLS measurements indicated that W-NPs were not only strongly agglomerated but also showed a high polydispersity in size. Current evidence suggested that agglomeration of nanoparticles is likely to affect their toxicity in biological systems. The outcomes observed in cell cultures may be influenced by factors that are only indirectly related to nanoparticles agglomeration, for example speed of sedimentation, diffusion coefficient or dissolution rates [38].

The results found in the current literature mainly proved that nano-sized particles of tungsten, alone as well as some combination-materials, seem to have a cytotoxic potential and induce apoptosis [12,13,19,20,39,40], mechanisms that were also identified in the present experimental system. Cell viability following exposure of W-NPs was evaluated by two complementary in vitro assays that assess cellular functions. Due to the ineffectiveness of correcting the interference between large nanoparticles and assay components, the MTT test was found unreliable in evaluating the toxicity of large W-NPs (100 nm). In the case of small NPs, MTT results indicated at 24 h a slight cytotoxic effect only at the highest concentration used (1 mg/mL). But the effect increased at 48 h, with viabilities significantly lower starting with 100 μg/mL.

Due to the fact that Vialight is known to be less prone to artifacts, faster and more sensitive, we considered it as an alternative method for cell viability assay, to overcome the interference of large W-NPs in the MTT assay. Vialight test showed that increasing concentrations of W-NPs of both sizes induced a decrease in cell viability. This effect was more pronounced for large W-NPs, with significant differences starting from 200 μg/mL. Overall, the cell viability seems to be more affected in the first 24 h of nanoparticles exposure, as compared with 48 h. These findings may indicate cytotoxicity with a certain threshold-like concentration-dependent pattern (100 μg/mL W-NPs), for both sizes of nanoparticles. Our result is in agreement with other investigations on W based NPs that identified a threshold NPs concentration of 100 μg/mL bellow which nanoparticles were found nontoxic [20,21,25].

In contradiction with most studies that indicate an inverse relation between particle size and toxicity, in our experimental conditions, the 100 nm W-NPs were more able to reduce the viability of BJ cells after 24 h, as compared to 30 nm W-NPs. A possible explanation could be that the toxic effects are not simply related to the primary size of NPs, the agglomeration state of W-NPs of 30 nm and 100 nm being likely to affect their toxicity. Our DLS data revealed that, at concentrations ≤100 μg/mL, the average agglomerate size of 30 nm W-NPs is larger than those formed by 100 nm W-NPs. Most probably, particle agglomeration and the deposition pattern of these agglomerates are influenced by different other factors including primary particle size [41].

The morphological changes of the cells following exposure to nanoparticles was evaluated by optical microscopy and SEM. There were observed cells with no severe morphological modifications or affected cells interconnectivity after 24 h exposure, even at high W-NPs concentrations. This is in close agreement with other study [20] that reported a similar structure of cells treated with tungsten oxide NPs, compared to the control cells. However, at higher concentrations, settlement of the W-NPs over the incubation time is observed, forming a layer that covers a large cell surface area (Supplementary material). Also, it was found that the particles could be internalized under the cell membrane, which can influence long-term cell viability. This statement is in accordance with our previous study [29] and also with findings by Ref. [13] where intracellular presence of NPs was observed to be restricted to the cytoplasm.

The present study was completed with the investigation of possible pathways of cell death, namely, necrosis (cell membrane integrity) and apoptosis (activation of caspases). By using membrane permeabilization-dependent Toxilight assay, it was found that the main pathway that induces BJ cell death following 24 h exposure to large W-NPs, especially at higher W-NPs concentrations, involves cell membrane damage. On the other hand, lack of a significant AK release observed after 48 h exposure, either to small or large W-NPs, would support the results obtained by the Vialight assay (based on the detection of cellular ATP which shows a relative viability increasement from 24 to 48 h, for concentrations ≥100 μg/mL. Good to notice that ATP increasement might have occurred in the absence of parallel increase of the number of viable cells, therefore this increasement may not fully sustain a survival mechanism [42].

Further, to determine if cells underwent apoptosis, we measured caspase 3/7 activity after 16 h of exposure to W-NPs. Caspases activation was found to significantly increase only for cells treated with small nanoparticles, at lower concentrations of W-NPs (1, 10, 100 μg/mL). At higher concentrations, caspases 3/7 may no longer remain active after 16 h of treatment, or possibly the apoptosis is induced by a pathway that is independent of caspases. Therefore, even if the cell's membrane integrity seems not to be affected after 24 h exposure to low concentrations of small NPs, at the same time cells were found prepared to undertake a programmed cell death (apoptosis). This result is in accordance with DLS measurement which highlighted lower concentrations (≤100 μg/mL) of small NPs having a microparticle behavior in a biological medium, due to the tendency to agglomerate. It is also in agreement with the observations from SEM images, which evidence some NPs presence on cell's body. This outcome is also in accordance with the Vialight outline because our results, showing that cytosolic ATP in cells is maintained at a higher level at these W-NPs concentrations, do not exclude the relevance of Caspase 3/7 activation after a 16 h exposure to the respective concentrations of large NPs. This high ATP level at low concentrations (<100 μg/ml), in contrast with the level observed at higher concentrations, is just a necessary condition to the apoptotic cell death process, most probably induced by apoptotic stimulation [43]. Therefore, we assume that the cells treated with 30 nm W-NPs, considering their high level of ATP, enter in an early stage of apoptosis, but, at that particular stage, the cytotoxicity evaluation and morphological changes are not yet concluded. Considering the multitude of pathways by which cell death can occur [44], which include extrinsic and intrinsic apoptosis pathways (ROS generation as an initiating factor of toxicity in nanomaterials exposed cells; lysosomal destabilization triggering the mitochondrial pathway of apoptosis), mitotic catastrophe or necrosis [45,46], the fact that we were able to evidence a fundamental difference on the impact of the nanoparticle size on the induced cytotoxicity and death pathway, is an important result, although other mechanisms involved remain to be further elucidated.

Besides our study, not much information is reported in the literature on the solubility of W containing particles, their oxidation following dispersion in biological media and their biological profile. Thus, the study [9] evaluated the chemical and colloidal stability of W particles in different media such as TRIS, LHC9, pulmonary culture medium and saline solution. It was observed that TRIS, LHC9 and pulmonary media did not strongly affect the average particle size, while dilution in saline leads to a substantial aggregation of the particles. With increasing time in suspension, W particles dissolved in all studied media [28]. W-NPs dissolution possibility has not been taken under study in our present work, hence additional tests are required in order to outline a full biological profile of W dust, in direct correlation with the verification at which time point cytotoxic effects are induced by ITER-like W-NPs and what is the highly toxic dimension that exists in the composition.

The present study findings have to be seen in light of some limitations, which could be addressed in future researches. First, focused on the MTT interference observed for large W-NPs, an alternative method that relies on cells metabolic activity should be used in future work to overcome this interference. Another limitation in our study is related to the mechanism of cell death. Even if we found a fundamental switch in the apoptotic response (pathway of caspase activation) triggered by low concentrations of small NPs and a significant and increasing AK release induced by higher concentration of large NPs, other exact mechanisms involved remain to be further elucidated. This assumption is based on the fact that different cell fates, including apoptosis and necrosis, are well known to be modulated by NPs physicochemical characteristics. The third limitation comes from membrane penetration by NP and internalization by cells. Besides our previous study [29], the present SEM images are just indicative of the NPs internalization, TEM images being needed to outline this process.

## 5 Conclusions

Despite its nowadays intensive use in the nanotechnology, engineering field [[47], [48], [49], [50], [51]] and in fusion technology, W involves a potential occupational or accidental risk. In particular, when thermonuclear fusion reactors, like ITER, become operational, W dust could represent a potential risk for environmental safety and for human health as they might be released in case of accidental situations, such as LOVA. But no conclusive information is yet available regarding the tungsten biological profile.

This study highlighted that ITER-like plasma nanoparticles, at different dimensions, have a cytotoxic effect on skin fibroblast BJ cell line and this effect is more pronounced for large nanoparticles (100 nm), at concentrations higher than 100 μg/mL. Moreover, we highlighted also that MTT assay is vulnerable to interference, due to the fact that large W-NPs, at a concentration of 100 μg/mL and higher, were found to reduce the tetrazolium compound in the MTT; however, it is not yet clear what role particle size play. Regarding the cell death mechanisms, we observed that the damage of the cell membrane is an important pathway by which BJ cell death occurs, more effectively when treated with higher concentrations of large nanoparticles. Apoptosis induced by caspases activation seems to be the main mechanism of BJ fibroblasts death that can be triggered by small W-NPs, but only at concentrations lower than 200 μg/ml. These different toxicological outputs identified as mechanistic responses of BJ cells after exposure to two different sizes of W-NPs are of critical importance in nanomaterial toxicity.

Therefore, the present study brings a necessary and useful progression in the characterization of the biological profile of ITER-released W-NPs, which may further support the assessment of potential risk that these nanoparticles, of different sizes, might pose on human health.

## Author contribution statement

Lavinia Gabriela Carpen, Ph.D. Student; Maria Adriana Acasandrei, Ph.D: Conceived and designed the experiments; Performed the experiments; Analyzed and interpreted the data; Wrote the paper.

Tomy Acsente, Ph.D.: Performed the experiments; Wrote the paper.

Elena Matei, Ph.D.; Iulia Lungu, Ph.D. Student: Performed the experiments; Contributed reagents, materials, analysis tools or data.

Gheorghe Dinescu, Ph.D.: Conceived and designed the experiments; Wrote the paper.

## Funding statement

This research was funded by the Romanian Ministry of Education and Research, Innovation and Digitization, and by the EUROfusion Consortium, funded by the European Union via the Euratom Research and Training Programme (Grant Agreement No 101052200 — EUROfusion).

## Data availability statement

Data will be made available on request.

## Declaration of interest's statement

The authors declare no competing interests.

## Additional information

Supplementary content related to this article has been published online at [URL].

## References

[1] Pitts R.A., Carpentier S., Escourbiac F., Hirai T., Komarov V., Lisgo S., Kukushkin A.S., Loarte A., Merola M., Sashala Naik A., Mitteau R., Sugihara M., Bazylev B., Stangeby P.C. (2013). A full tungsten divertor for ITER: physics issues and design status. J. Nucl. Mater..

[2] Grisolia C., Gensdarmes F., Peillon S., Dougniaux G., Bernard E., Autricque A., Pieters G., Rousseau B., Feuillastre S., Garcia-Argote S., Carvalho O., Malard V., George I., Lebaron-Jacobs L., Orsiere T., Uboldi C., Rose J., Sanles Sobrido M., Lambertin D., Vrel D., Decanis C., Liger K. (2019). Current investigations on tritiated dust and their impact on tokamak safety. Nucl. Fusion.

[3] Sharpe J.P., Petti D.A., Bartels H.-W. (2002). A review of dust in fusion devices: implications for safety and operational performance. Fusion Eng. Des..

[4] McCarthy K.A., Petti D.A., Carmack W.J., Smolik G.R. (1998). The safety implications of tokamak dust size and surface area. Fusion Eng. Des..

[5] Federici G., Skinner C.H., Brooks J.N., Coad J.P., Grisolia C., Haasz A.A., Hassanein A., Philipps V., Pitcher C.S., Roth J., Wampler W.R., Whyte D.G. (1967). Plasma-material interactions in current tokamaks and their implications for next step fusion reactors. Nucl. Fusion.

[6] Taylor N., Cortes P. (2014). Lessons learnt from ITER safety & licensing for DEMO and future nuclear fusion facilities. Fusion Eng. Des..

[7] Ivanova D.-D. (2012).

[8] Malizia A., Antonio Poggi L., Ciparisse J.-F., Rossi R., Bellecci C., Gaudi P. (2016). A review of dangerous dust in fusion reactors: from its creation to its resuspension in case of LOCA and LOVA. Energies.

[9] Sobrido M., Bernard E., Angeletti B., Malard V., George I., Chaurand P., Uboldi C., Orsière T., Dine S., Vrel D., Rousseau B., Dinescu G., Soulas R., Herlin N., Proux O., Grisolia C., Rose J. (2020). Oxidative transformation of Tungsten (W) nanoparticles potentially released in aqueous and biological media in case of Tokamak (nuclear fusion) Lost of Vacuum Accident (LOVA). C.R. Geosci..

[10] Bernard E., Jambon F., Georges I., Sanles Sobrido M., Rose J., Herlin-Boime N., Miserque F., Beaunier P., Vrel D., Dine S., Hodille E., Chêne J., Garcia-Argote S., Pieters G., Peilloni S., Gensdarmes F., Dinescu G., Acsente T., Uboldi C., Orsiere T., Malard V., Rousseau B., Delaporte Ph, Grisolia C. (2019). Design of model tokamak particles for future toxicity studies: morphology and physical characterization. Fusion Eng. Des..

[11] Uboldi C., Sanles Sobrido M., Bernard E., Tassistro V., Herlin-Boime N., Vrel D., Garcia-Argote S., Roche S., Magdinier F., Dinescu G., Malard V., Lebaron-Jacobs L., Rose J., Rousseau B., Delaporte P., Grisolia C., Orsière T. (2019). In vitro analysis of the effects of ITER-like tungsten nanoparticles: cytotoxicity and epigenotoxicity in BEAS-2B cells. Nanomaterials.

[12] Armstead A.L., Arena C.B., Li B. (2014). Exploring the potential role of tungsten carbide cobalt (WC-Co) nanoparticle internalization in observed toxicity toward lung epithelial cells in vitro. Toxicol. Appl. Pharmacol..

[13] Bastian S., Busch W., Kühnel D., Springer A., Meißner T., Holke R., Scholz S., Iwe M., Pompe W., Gelinsky M. (2009). Toxicity of tungsten carbide and cobalt-doped tungsten carbide nanoparticles in mammalian cells in vitro. Environ. Health Perspect..

[14] Moche H., Chevalier D., Barois N., Lorge E., Claude N., Nesslany F. (2014). Tungsten carbide-cobalt as a nanoparticulate reference positive control in in vitro genotoxicity assays. Toxicol. Sci..

[15] Lanone S., Rogerieux F., Geys J., Dupont A., Maillot-Marechal E., Boczkowski J., Lacroix G., Hoet P. (2009). Comparative toxicity of 24 manufactured nanoparticles in human alveolar epithelial and macrophage cell lines. Part. Fibre Toxicol..

[16] WHO International Agency for Research on Cancer (2006).

[17] Paget V., Moche H., Kortulewski T., Grall R., Irbah L., Nesslany F., Chevillard S. (2015). Human cell line-dependent WC-Co nanoparticle cytotoxicity and genotoxicity: a key role of ROS production. Toxicol. Sci..

[18] Osterburg A.R., Robinson C.T., Schwemberger S., Mokashi V., Stockelman M., Babcock G.F. (2010). Sodium tungstate (Na2WO4) exposure increases apoptosis in human peripheral blood lymphocytes. J. Immunot..

[19] Laulicht F., Brocato J., Cartularo L., Vaughan J., Wu F., Kluz T., Sun H., Oksuz B.A., Shen S., Medici S., Zoroddu M.A., Costa M., Peana M. (2015). Tungsten-induced carcinogenesis in human bronchial epithelial cells. Toxicol. Appl. Pharmacol..

[20] Chinde S., Poornachandra Y., Panyala A., Kumari S.I., Yerramsetty S., Adicherla H., Grover P. (2018). Comparative study of cyto-and genotoxic potential with mechanistic insights of tungsten oxide nano- and microparticles in lung carcinoma cells: genotoxic potential of tungsten oxide nanoparticles in A549 cells. J. Appl. Toxicol..

[21] Turkez H., Sonmez E., Turkez O., Mokhtar Y.I., Di Stefano A., Turgut G. (2014). The risk evaluation of tungsten oxide nanoparticles in cultured rat liver cells for its safe applications in nanotechnology. Braz Arch Biol.

[22] Akbaba B.G., Turkez H., Sonmez E., Akbaba U., Aydın Karataş E., Tatar A., Turgut G., Çeriğ S. (2016). In vitro genotoxicity evaluation of tungsten (VI) oxide nanopowder using human lymphocytes. Biomed. Res..

[23] Jain K., Kohli E., Prasad D., Kamal K., Haussain S.M., Singh S.B. (2014). In vitro cytotoxicity assessment of metal oxide nanoparticle. Nanomedicine and Nanobiology.

[24] Ivask A., Titma T., Visnapuu M., Vija H., Käkinen A., Sihtmäe M., Pokhrel S., Madler L., Heinlaan M., Kisand V., Shimmo R., Kahru A. (2015). Toxicity of 11 metal oxide nanoparticles to three mammalian cell types in vitro. Curr. Top. Med. Chem..

[25] Hussain S.M., Hess K.L., Gearhart J.M., Geiss K.T., Schlager J. (2005). J. In vitro toxicity of nanoparticles in BRL 3A rat liver cells. Toxicol. Vitro.

[26] Bolt A.M., Sabourin V., Molina M.F., Police A.M., Negro Silva L.F., Plourde D., Lemaire M., Ursini-Siegel J., Mann K.K. (2015). Tungsten targets the tumor microenvironment to enhance breast cancer metastasis. Toxicol. Sci..

[27] Machado B., Suro R.M., Garza K.M., Murr L. (2011). Comparative microstructures and cytotoxicity assays for ballistic aerosols composed of micrometals and nanometals: respiratory health implications. Int. J. Nanomed..

[28] George I., Uboldi C., Bernard E., Sobrido M.S., Dine S., Hagège A., Vrel D., Herlin N., Rose J., Orsière T., Grisolia C., Rousseau B., Malard V. (2019). Toxicological assessment of ITER-like tungsten nanoparticles using an in vitro 3D human airway epithelium model. Nanomaterials.

[29] Carpen L.G., Acsente T., Acasandrei M.A., Matei E., Chilom C.G., Savu D.I., Dinescu G., Clichici S., Filip A., Gustavo M. (2019). Nanomaterials - Toxicity, Human Health and Environment.

[30] Acsente T., Negrea R.F., Nistor L.C., Logofatu C., Matei E., Birjega R., Grisolia C., Dinescu G. (2015). Synthesis of flower-like tungsten nanoparticles by magnetron sputtering combined with gas aggregation. Eur. Phys. J. D.

[31] Acsente T., Negrea R.F., Nistor L.C., Matei E., Grisolia C., Birjega R., Dinescu G. (2017). Tungsten nanoparticles with controlled shape and crystallinity obtained by magnetron sputtering and gas aggregation. Mater. Lett..

[32] (2021). ImageJ.

[33] Oancea M., Mazumder S., Crosby M.E., Almasan A. (2006). Apoptosis assays. Methods Mol. Med..

[34] Crowley L.C., Marfell B.J., Waterhouse N.J. (2016). Analyzing cell death by nuclear staining with Hoechst 33342. Cold Spring Harb. Protoc..

[35] Baig N., Kammakakam I., Falathabe W. (2021). Nanomaterials: a review of synthesis methods, properties, recent progress, and challenges. Mater. Adv..

[36] El-Eskandarany M.S., Al-Hazza A., Al-Hajji L.A., Ali N., Al-Duweesh A.A., Banyan M., Al-Ajmi F. (2021). Mechanical milling: a superior nanotechnological tool for fabrication of nanocrystalline and nanocomposite materials. Nanomaterials.

[37] Ozolin A.V., Sokolov E.G., Golius D.A. (2020). Obtaining of tungsten nanopowders by high energy ball milling. IOP Conf. Ser. Mater. Sci. Eng..

[38] Bruinink A., Wang J., Wick P. (2015). Effect of particle agglomeration in nanotoxicology. Arch. Toxicol..

[39] Lombaert N., De Boeck M., Decordier I., Cundari E., Lison D., Kirsch-Volders M. (2004 Dec 1). Evaluation of the apoptogenic potential of hard metal dust (WC-Co), tungsten carbide and metallic cobalt. Toxicol. Lett..

[40] Lombaert N., Lison D., Van Hummelen P., Kirsch-Volders M. (2008 Mar 1). In vitro expression of hard metal dust (WC-Co)--responsive genes in human peripheral blood mononucleated cells. Toxicol. Appl. Pharmacol..

[41] Gosens I., Post J.A., de la Fonteyne L.J., Jansen E.H., Geus J.W., Cassee F.R., de Jong W.H. (2010). Impact of agglomeration state of nano-and submicron sized gold particles on pulmonary inflammation. Part. Fibre Toxicol..

[42] Scholfield C., Simmons T., Gill S., Olsen C. (1993). Cytotoxicity in cells: easy determination using Lonza ViaLight plus and ToxiLight BioAssays on the SpectraMax L microplate luminometer. Molecular Devices.

[43] Zamaraeva M.V., Sabirov R.Z., Maeno E., Ando-Akatsuka Y., Bessonova S.V., Okada Y. (2005). Cells die with increased cytosolic ATP during apoptosis: a bioluminescence study with intracellular luciferase. Cell Death Differ..

[44] Elbadawi G.Y.M., Efferth T. (2020). Multiple cell death modalities and their key features (Review). World Academy of Sciences Journal.

[45] De Stefano D., Carnuccio R., Maiuri M. (2012). C nanomaterials toxicity and cell death modalities. J Drug Deliv.

[46] Yang Y., Du X., Wang Q., Liu J., Zhang E., Sai L., Peng C., Lavin M.F., Yeo A.J., Yang X., Shao H., Du Z. (2019). Mechanism of cell death induced by silica nanoparticles in hepatocyte cells is by apoptosis. Int. J. Mol.

[47] Joy J., Mathew J., George S.C. (2018). Nanomaterials for photoelectrochemical water splitting – review. Int. J. Hydrogen Energy.

[48] Zhu T., Meng D., Chong N., Chan S. (2014). Nanostructured tungsten trioxide thin films synthesized for photoelectrocatalytic water oxidation: a review. ChemSusChem.

[49] Yin Z., Bu Y., Ren J., Chen S., Zhao D., Zou Y., Shen S., Yang D. (2018). Triggering superior sodium ion adsorption on (2 0 0) facet of mesoporous WO3 nanosheet arrays for enhanced supercapacitance. Chem. Eng. Sci..

[50] Han Z., Golev A., Edraki M.A. (2021). Review of tungsten resources and potential extraction from mine waste. Minerals.

[51] Carpen L.G., Acsente T., Sătulu V., Matei E., Vizireanu S., Bita B.I., Dinescu G. (2021). Hybrid nanostructures obtained by transport and condensation of tungsten oxide vapours onto CNW templates. Nanomaterials.

