# Supplementary material for: In vitro analysis of the cytotoxic effect of two different sizes ITER-like tungsten nanoparticles on human dermal fibroblasts
Source: Heliyon. 2023 Feb 18;9(3):e13849. doi: 10.1016/j.heliyon.2023.e13849 (PMC9988585; doi:10.1016/j.heliyon.2023.e13849)
Supplement: Supplementary_Material [file mmc1.docx]

In vitro analysis of the cytotoxic effect of two different sizes ITER-like tungsten nanoparticles on human dermal fibroblasts

Lavinia Gabriela Carpen, Maria Adriana Acasandrei, Tomy Acsente, Elena Matei, Iulia Lungu, Gheorghe Dinescu

**Supplementary Materials**

**Morphology information of biological samples analyzed by SEM imaging**

The following SEM images were taken by using Gemini 500 equipment from Zeiss (Oberkochen, Germany) and they reflect the morphological changes in the treated samples as compared to the controls. The Gemini 500 equipment allowed us to analyze aspects of cells morphology without gold coating, in order not to alter or hide the structure of the basic sample, which is very important especially in the case of small nanoparticles. On the other hand, by using Gemini 500 equipment we observed W NPs behavior only on the cell surface. We could not perform an investigation under the cell membrane, to conform that the nanoparticles, initially adhered to the cell membrane, could be subsequently internalized by cells.

**Control Sample**

The images taken from the control sample show the normal morphology of the fibroblasts cells. It can be seen how the cells tend to cover the entire surface on which they were previously inoculated. An important aspect, emphasized at large magnification, is that cells are tightly interconnected, suggesting the cells have a normal development.


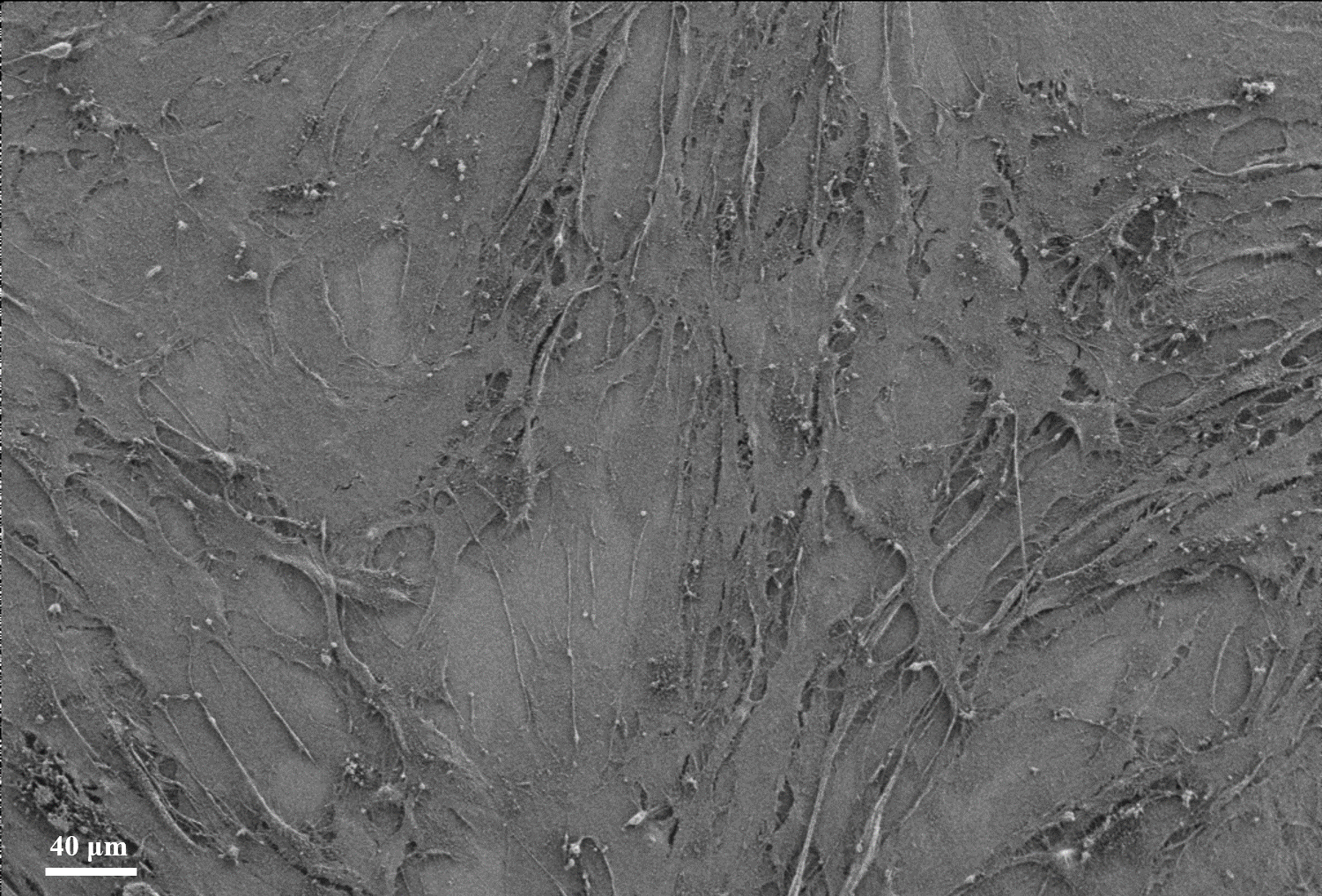


Figure A1. SEM images that reveal the morphology of the untreated BJ cells (control).

**Treated Samples – Cell morphological observations when W NPs were used**

**C1 (1 µg/mL)**

| **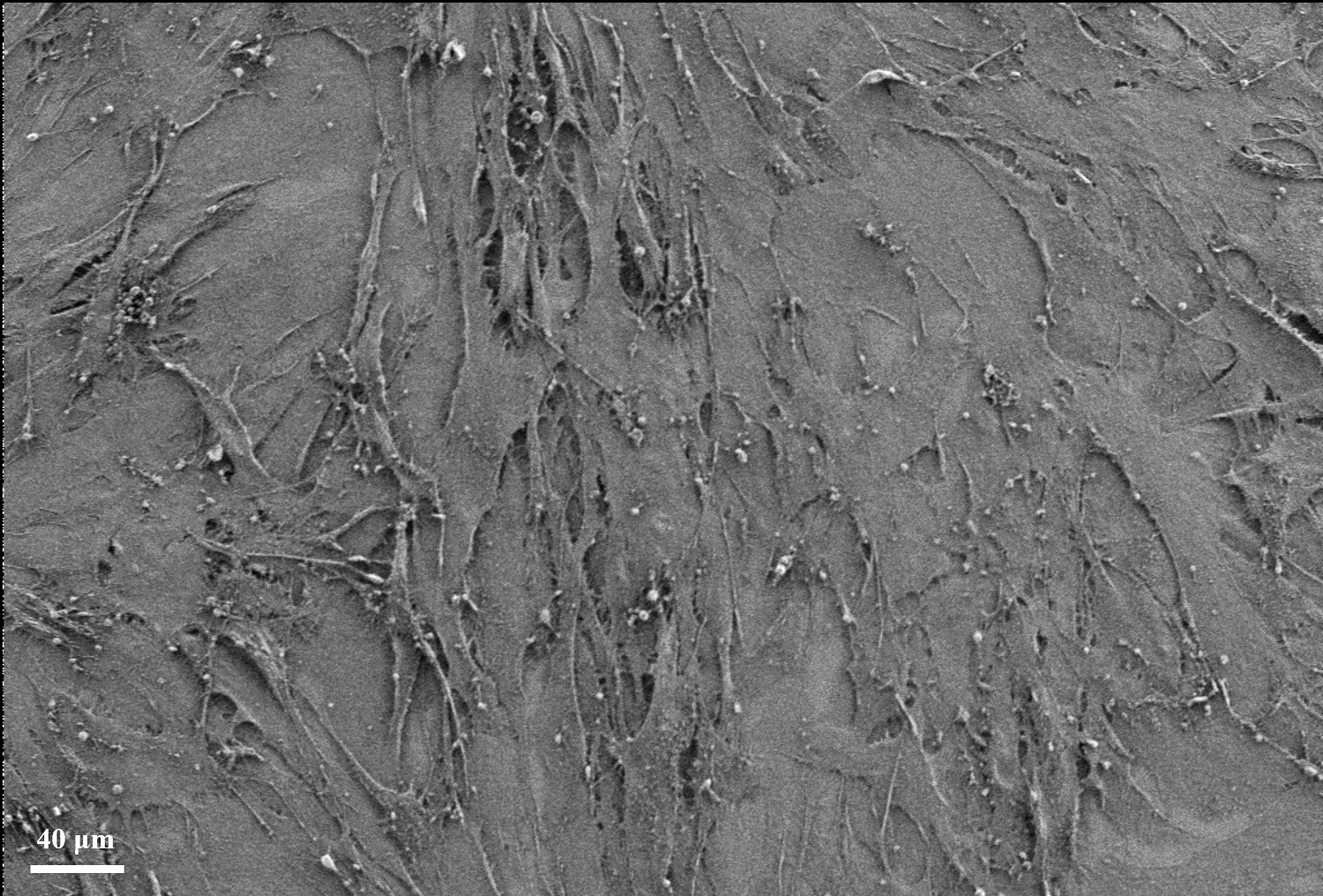** | **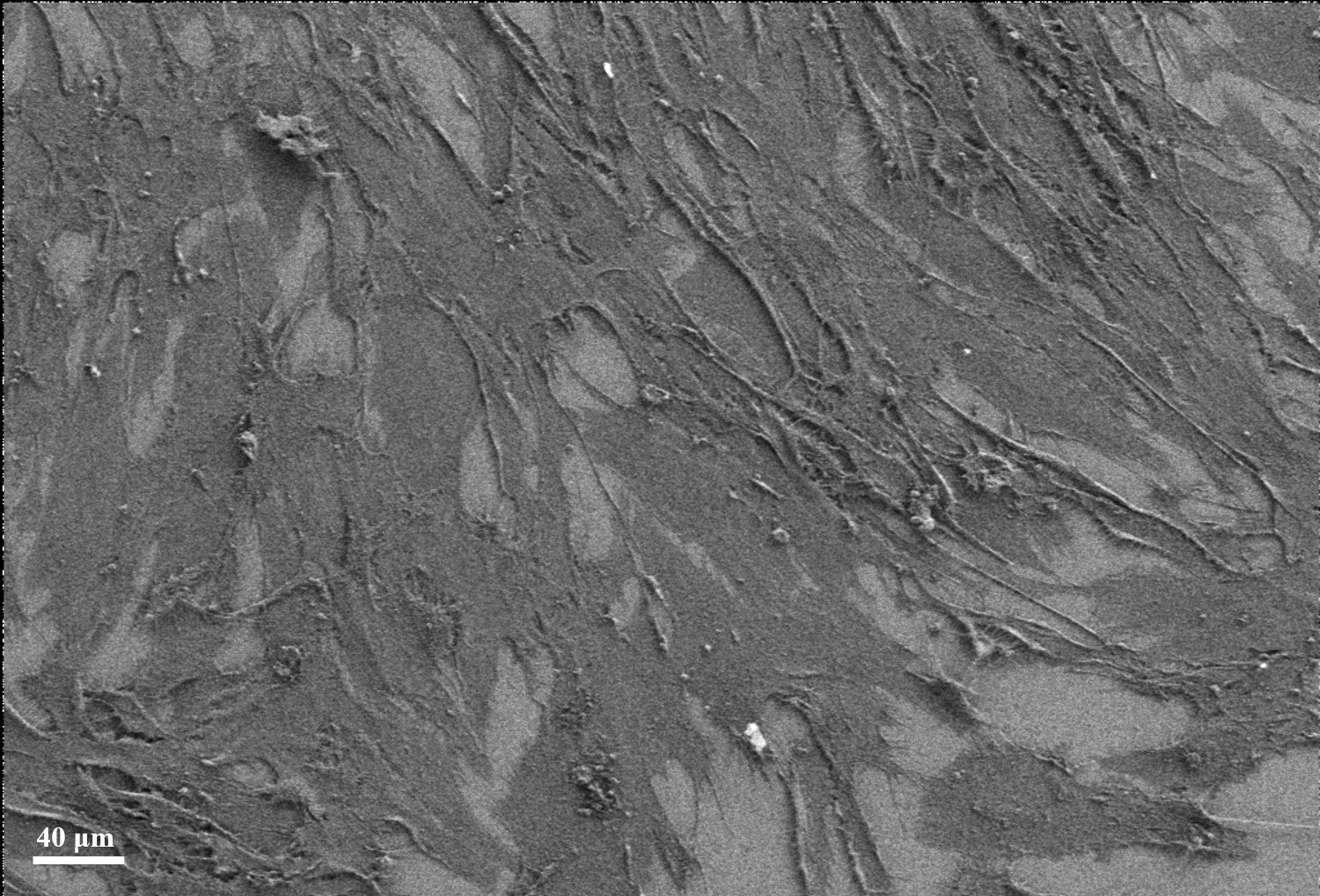** |
| --- | --- |
| Figure A2. SEM images that reveal the cells morphology after being treated with 1 µg/mL of small W NPs (c1) | Figure A3. SEM images that reveal the cells morphology after being treated with 1 µg/mL of large W NPs (c1) |


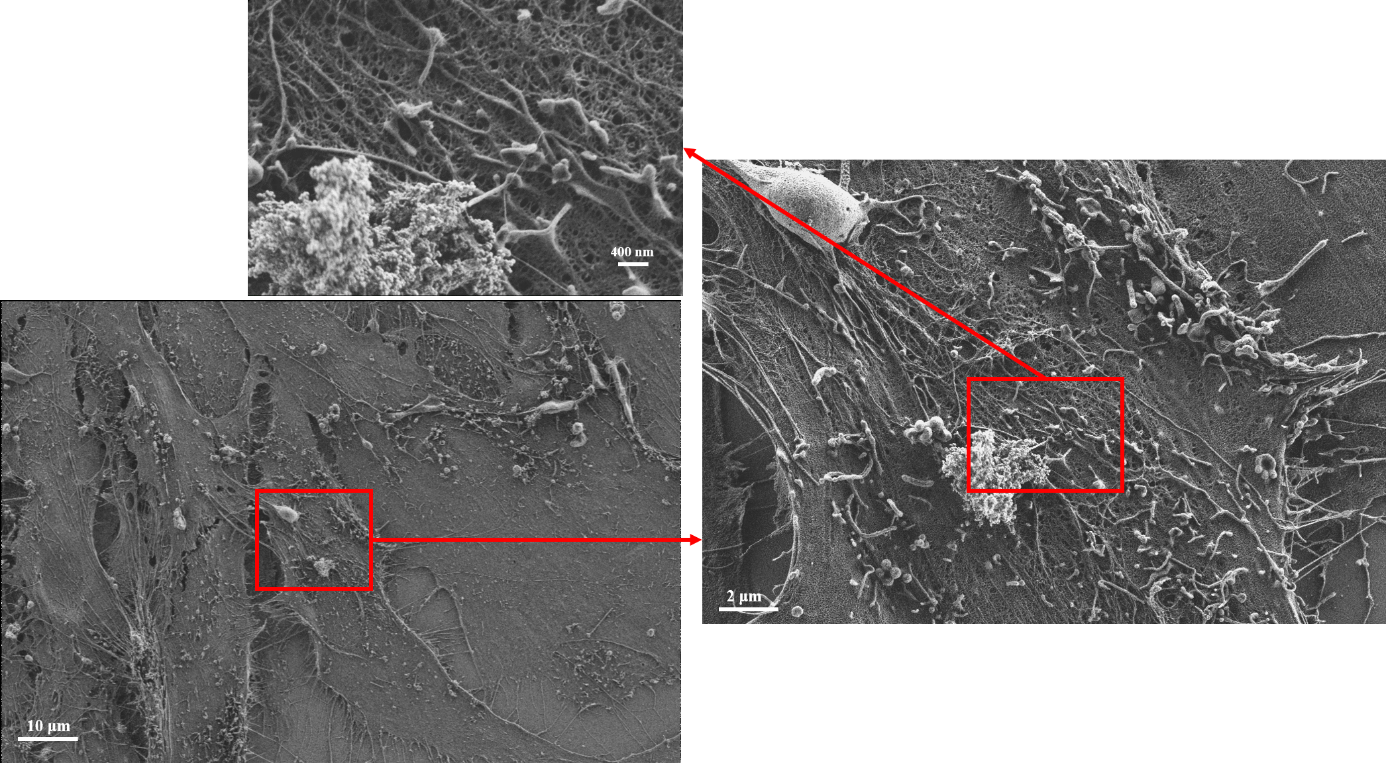
Figure A4. SEM images, at different magnifications, which reveal the cells morphology and addesion of agglomerated W NPs on cell’s surface, after being treated with1 µg/mL of small W NPs (c1)


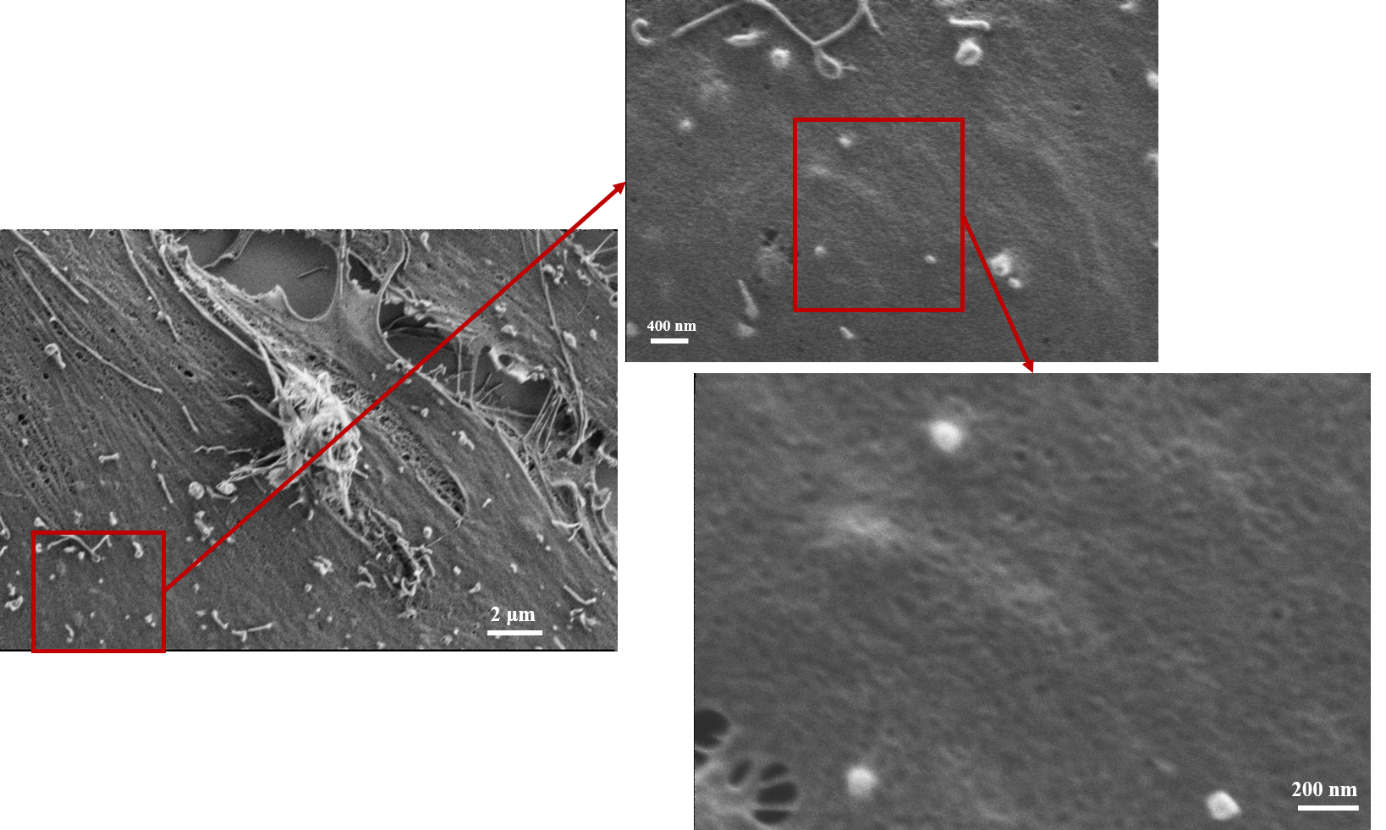


Figure A5. SEM images, at different magnifications, which reveal the cells morphology after being treated with 1 µg/mL of large W NPs (c1).

At low W NPs concentrations (<10 μg / mL) added over the initially inoculated cells, it is difficult to observe the presence of individual W NPs on the cell body surface. In the case of large NPs, more individual particles are observed, while the tendency of agglomeration is noticeable especially in the case of small NPs.

**C3 (100 µg/mL)**

| 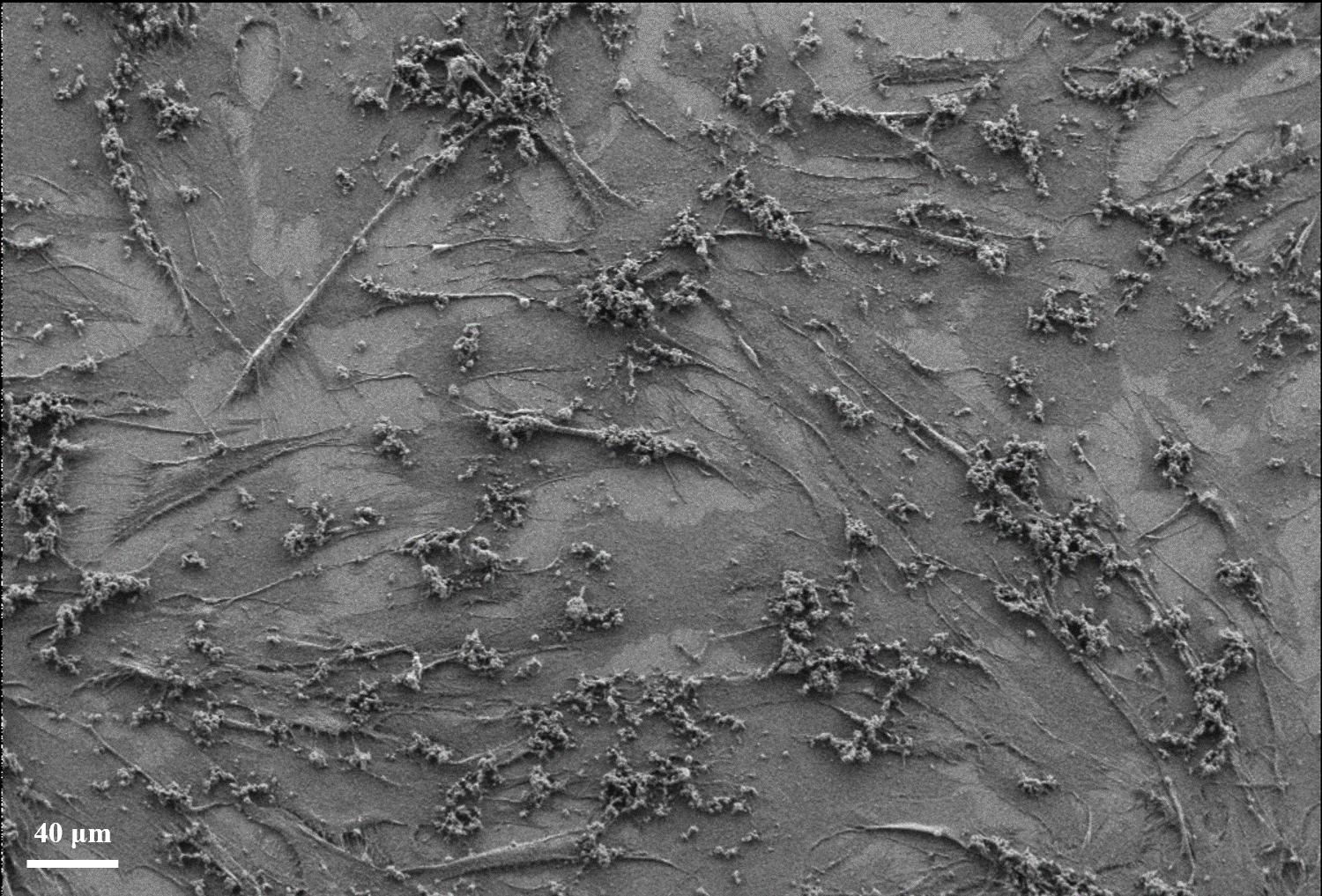 | 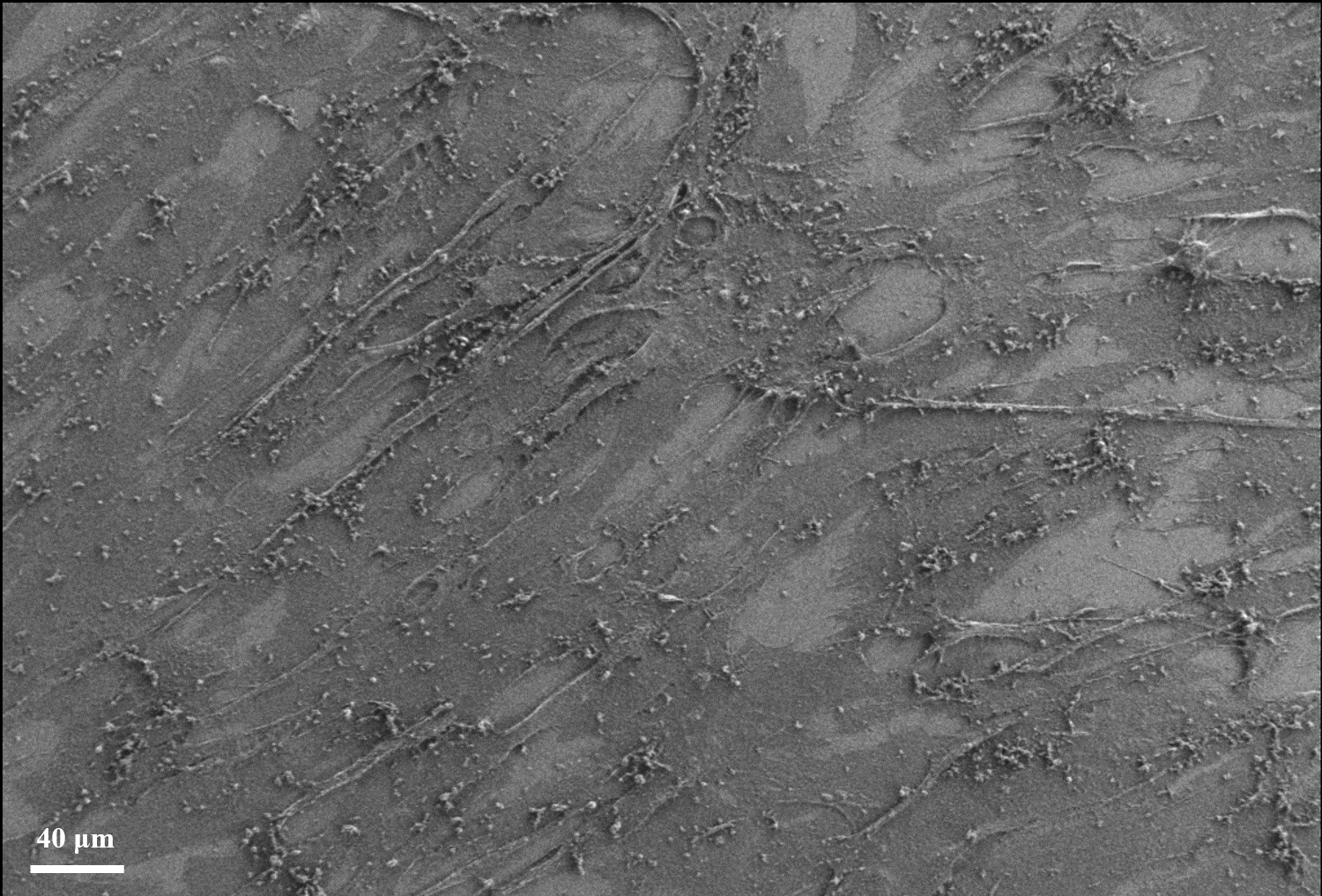 |
| --- | --- |
| a) | a) |
| 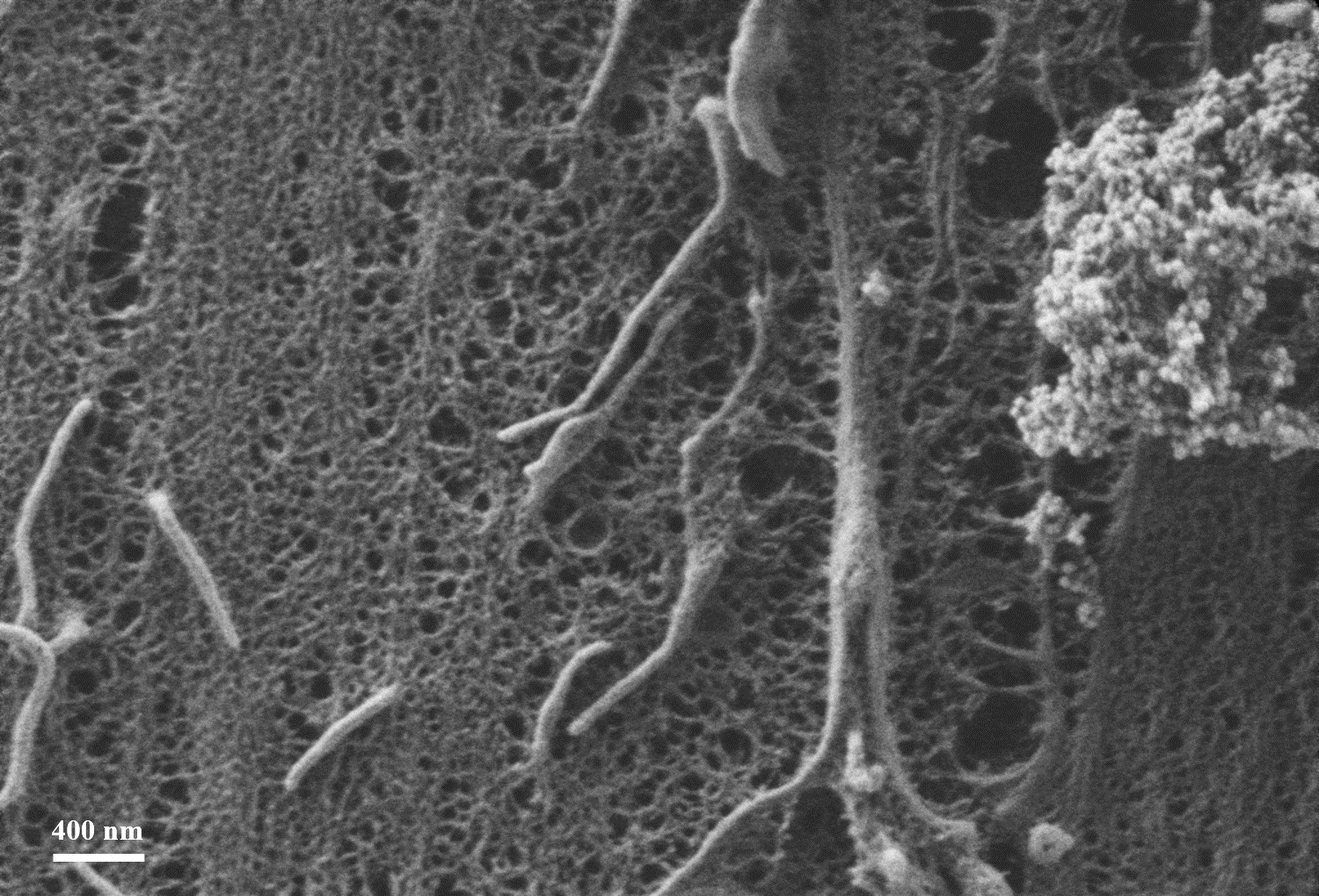 | 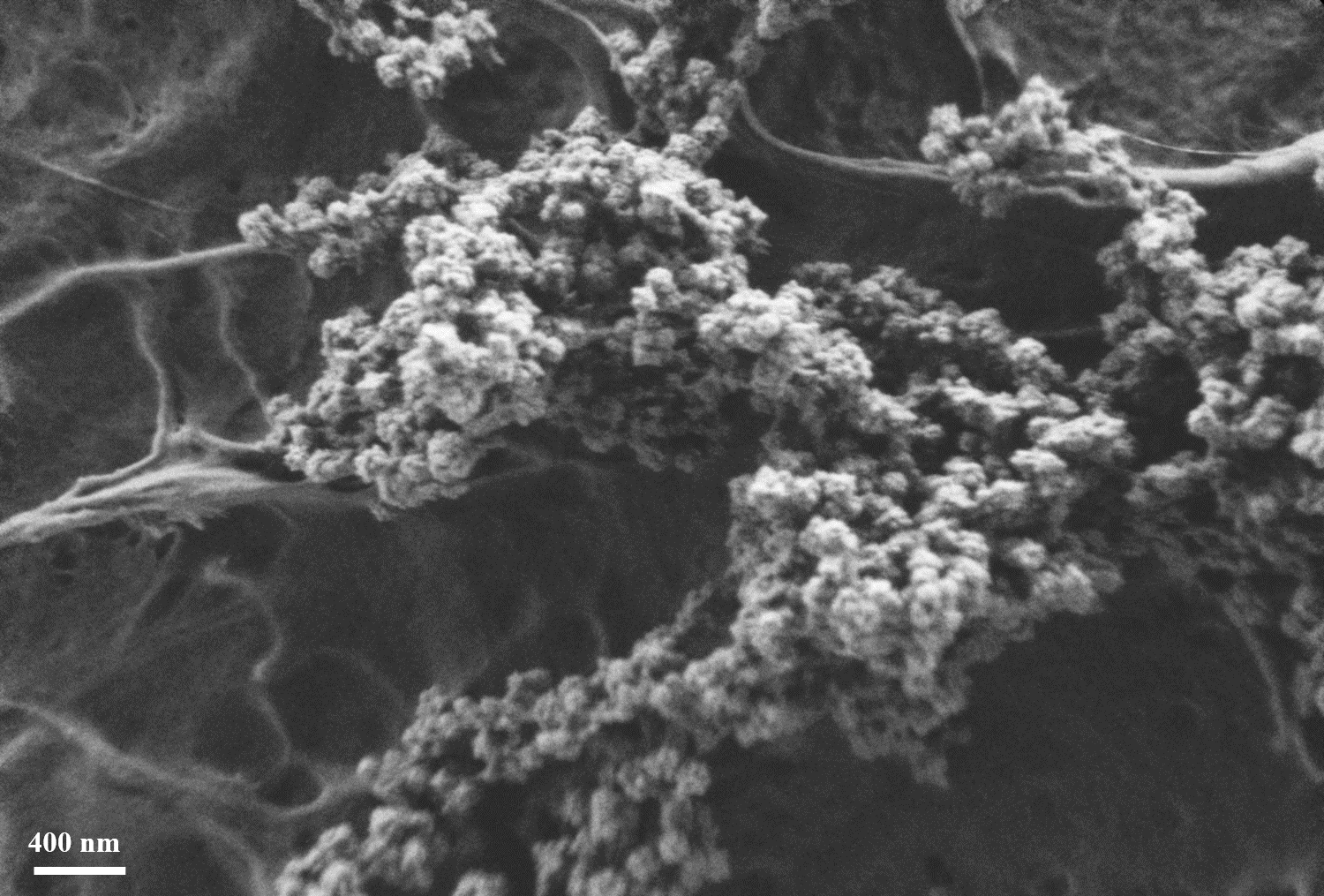 |
| b) | b) |
| Figure A6. SEM images that reveal cells morphology after small W NPs (c3 concentration) interact with BJ cells, at different magnifications (a and b). | Figure A7. SEM images that reveal cells morphology after large W NPs (c3 concentration) interact with BJ cells, at different magnifications (a and b). |

At 100 μg / mL (c3) of tungsten nanoparticles added over the initially inoculated cells, the tendency of the nanoparticles to agglomerate and how these nanoparticle agglomerations cover part of the cell body are highlighted. However, cell morphology is not affected, neither the cells interconnections.

**C6 (1000 µg/mL)**

| 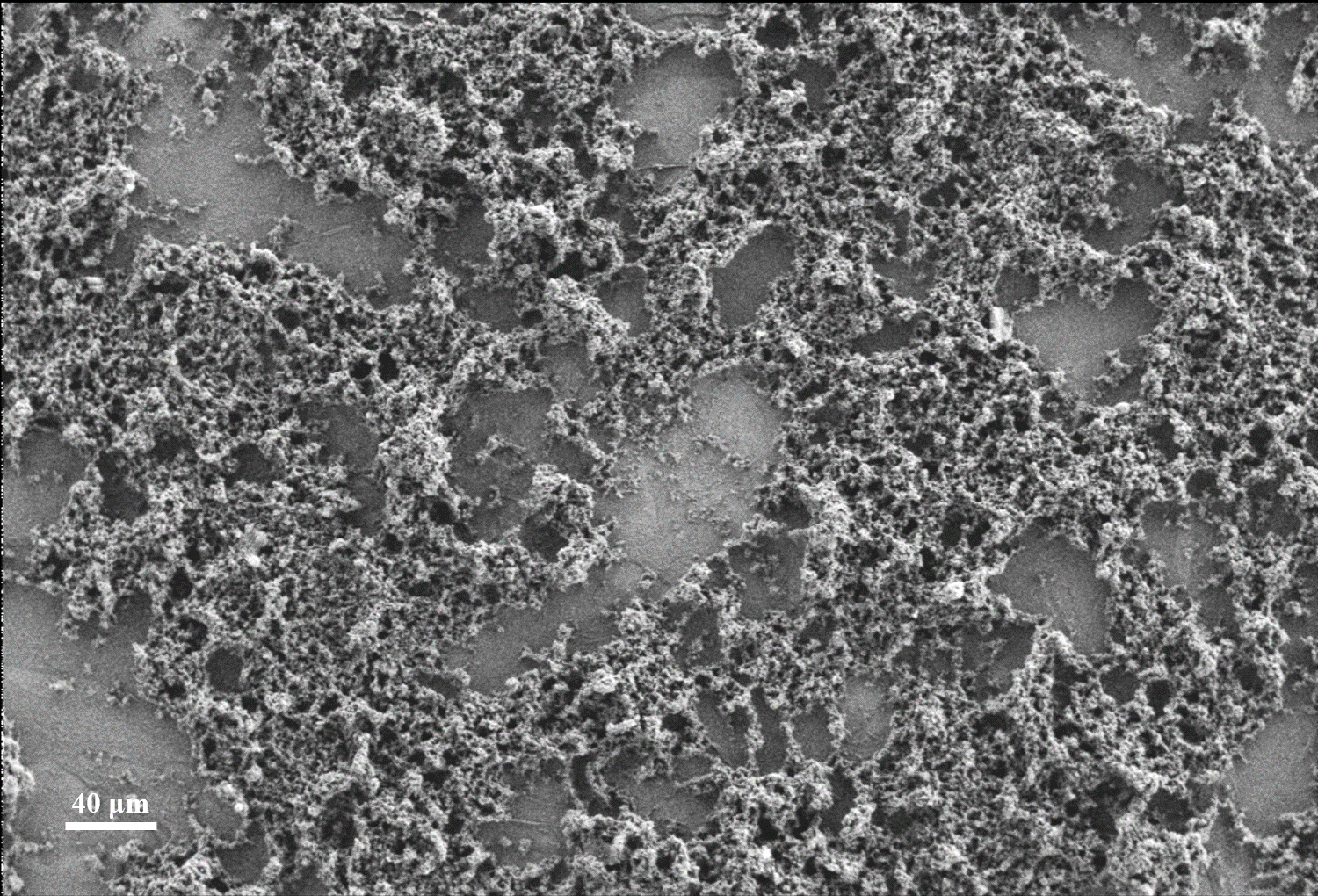 | 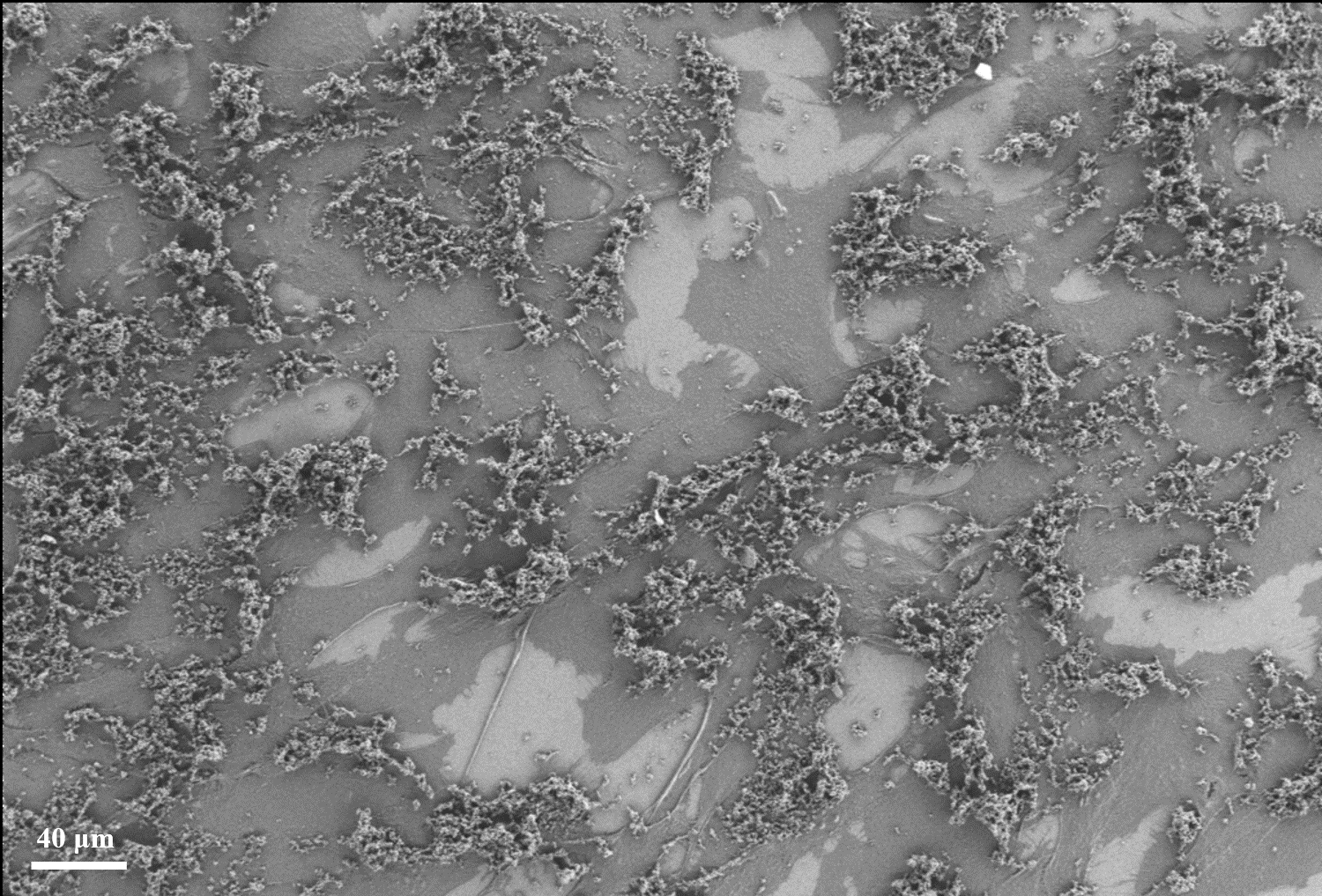 |
| --- | --- |
| Figure A8. SEM images that reveal the morphology changes after small W NPs (c6 concentration) interact with BJ cells. | Figure A9. SEM images that revel the morphology changes after large W NPs (c6 concentration) interact with BJ cells. |

When using high concentrations of nanoparticles (c6 - 1000 µg/mL) it is observed that the agglomerates settled and covered the entire surface on which the cells are anchored. This is more evident in the case of small nanoparticles.

By comparing these two samples (small and large nanoparticles) at low magnifications, it is observed that the level of cells distribution on the surface is lower when the nanoparticles concentration added in cell medium is increased. However, there is no major difference in cell development and, implicitly, the morphology of the cells compared to the control sample, which indicates that, after 24 hours of treatment, cells morphology is not severely affected by this exposure.
